# Clinical and Gut Microbiome Characteristics of Medically Complex Patients Receiving Blenderized Tube Feeds vs. Standard Enteral Feeds †

**DOI:** 10.3390/nu17122018

**Published:** 2025-06-17

**Authors:** Marianelly Fernandez Ferrer, Mauricio Retuerto, Aravind Thavamani, Erin Marie San Valentin, Thomas J. Sferra, Mahmoud Ghannoum, Senthilkumar Sankararaman

**Affiliations:** 1 Division of Pediatric Gastroenterology, Hepatology & Nutrition, Department of Pediatrics, UH Rainbow Babies and Children’s Hospital, Cleveland, OH 44106, USA; mcfernandezferrer@gmail.com (M.F.F.); aravind.thavamani@uhhospitals.org (A.T.); thomas.sferra@uhhospitals.org (T.J.S.); 2 Department of Pediatrics, Case Western Reserve University School of Medicine, Cleveland, OH 44106, USA; 3 Center for Medical Mycology, Department of Dermatology, Case Western Reserve University School of Medicine, Cleveland, OH 44106, USA; retuertomauricio@gmail.com (M.R.); exs785@case.edu (E.M.S.V.); mag3@case.edu (M.G.); 4 Department of Dermatology, University Hospitals Cleveland Medical Center, Cleveland, OH 44106, USA

**Keywords:** gut microbiome, microbiota, blended diet, blenderized tube feeds, enteral feeding, dysbiosis

## Abstract

**Background:** Diet is known to influence the composition of the gut microbiome. For patients who require enteral feeding, there has been a growing popularity of using blenderized tube feeds (BTFs) as an alternative to standard enteral formula (SEF). There is limited literature exploring the impact of BTFs on the gut microbiome. **Methods:** Twenty-eight patients 1 to 22 years of age who received their nutrition via gastrostomy tube for over 4 weeks were included and participants were divided into BTF and SEF groups. Demographics and clinical information were collected from the medical records, and all legal guardians completed a semi-structured interview using a questionnaire. 16SrRNA sequencing was used for bacteriome analysis. **Results:** Eleven patients in the BTF group and seventeen in the SEF group were included. No significant differences in the demographics were noted. Patients on BTFs had no emesis compared to seven (41%) in the SEF group, *p* = 0.02. There were no significant differences in other clinical characteristics and comorbidities. No significant differences in the gut microbiome between the groups were noted for alpha and beta diversities, richness, and evenness (at both genus and species levels). Differential abundance analysis showed only a few significant differences between the groups at all reported taxonomic levels. **Conclusions:** Patients on BTFs had a significantly decreased prevalence of emesis compared to the SEF group. No significant differences in the microbiome between the groups were noted for alpha and beta diversities, richness, and evenness. Prospective studies are recommended to verify our preliminary data and further evaluate the implications of our study results.

## 1. Introduction

Children with medical complexity often experience gastrointestinal (GI) intolerance symptoms at a higher rate and severity [1]. Enteral nutrition provided directly to the stomach or small bowel, bypassing the oral cavity, is necessary for these patients when their oral nutrition intake is insufficient or unsafe [2,3,4,5]. Standard, complete, commercially prepared, mostly dairy-based formulas (liquid or powdered preparations) are considered the accepted approach for enteral nutrition [6]. These standard enteral formula (SEF) products include polymeric with intact protein (dairy-based or soy-based), extensively hydrolyzed (oligomeric peptide-based), or elemental (amino acid-based) formulas [7].

GI intolerance in these patients often leads to frequent healthcare utilization and adversely impacts the quality of life of both patients and their family members [4,8,9]. For patients with GI intolerance, there has been increasing interest in the utility of blenderized tube feedings (BTFs). BTFs refer to commercial formulae manufactured with whole food ingredients or homemade liquidized whole food-based blenderized foods [6,10,11,12,13,14,15]. In 2011, Pentiuk and colleagues noted a reduction in gagging and retching behaviors in 33 post-fundoplication children after transitioning to a pureed blenderized diet via gastrostomy tube [11].

Children with medical complexity were noted to have dysbiosis with lower alpha diversity indices and less abundant butyric acid-producing bacteria compared to healthy controls [16]. Diet is known to strongly influence the gut microbiome [17,18,19,20]. A study including fecal samples from 91 children showed a significant decrease in alpha diversity in children with medical complexity receiving exclusive enteral therapy [9]. Further understanding of the impact of different enteral nutrition alternatives on the gut microbiome could lead to approaches to modulate the gut microbiome and address GI symptoms in medically complex patients.

The literature exploring the impact of BTFs on symptoms of GI intolerance and the gut microbiome is limited, and we sought to investigate this further [21,22]. We hypothesized that patients on BTF would have decreased GI symptoms and increased alpha diversity when compared to patients on the SEF. Our primary aim was to evaluate the prevalence of GI symptoms in patients on BTF and compare them with the SEF group. The secondary aim was to compare the characteristics of the gut microbiome between BTF and SEF groups.

## 2. Materials and Methods

### 2.1. Study Design

This single-center, prospective, observational study was performed between January 2023 and May 2024.

### 2.2. Inclusion Criteria

Both outpatient clinic schedules and inpatient admission lists of the pediatric gastroenterology division were screened regularly for potential participants above one year of age receiving more than 75% of the nutrition via gastrostomy tube. Patients receiving BTFs (commercial or homemade or a combination of both, also referred to as cases in the figures) or SEF (polymeric or protein-hydroxylate or elemental formulas, also referred to as the control group in the figures) for a duration of at least 4 weeks were considered eligible for inclusion.

### 2.3. Exclusion Criteria

Patients who had recently used antimicrobials in the past two weeks (except for erythromycin as a promotility agent), GI infections in the preceding four weeks, short gut syndrome, or mucosal diseases such as inflammatory bowel disease were excluded. Patients on acid suppression medications, laxatives, and probiotics were not excluded as these medications are commonly prescribed in the selected patient population.

All legal guardians signed their informed consent. Demographic and clinical information was obtained from the medical records, and all guardians completed a semi-structured interview (Supplemental Document) using a questionnaire administered by the primary investigator at the time of enrollment. The initiation of BTFs vs. SEF was decided by their primary pediatric gastroenterologist and the nutrition team and was not influenced by the research team.

### 2.4. Sample Size Estimation

Prior studies have shown significant differences (*p* < 0.05) in alpha diversity between homemade BTF vs. ready-to-use feeds in a small sample (6 vs. 5) of children [21]. Another systematic review compiled 11 studies and detailed the microbiome differences between patients on various nutritional interventions (enteral nutrition vs. parenteral nutrition). Here, the sample sizes varied between 7 and 42 in the study population vs. 5–40 in the control groups [23]. Taking these factors into account, we aimed to recruit at least 10 patients in each group for this exploratory study.

### 2.5. Sample Collection and Processing

The samples were collected by parents/legal guardians, who were provided with a collection kit and specific written instructions during enrollment, or by the research team during the clinic visits. A stool sample kit (BD BBL™ CultureSwab™ EZ, Franklin Lakes, NJ, USA) was provided, along with instructions, to the participant’s families. Stool specimens were collected and stored in a consistent way to minimize confounding effects. All collected stool samples were instantly placed in FastPrep^®^ tubes (MP Biomedicals™, Cat# 5076-200-34340, Solon, OH, USA) containing 500 μL of glass beads (Sigma-Aldrich G8772-100g, St. Louis, MO, USA) and 1 mL ASL ™ lysis buffer (Qiagen DNA Extraction Kit, Hilden, Germany). To minimize the batch effect, all samples were stored at −20 °C, then processed and analyzed concurrently for microbial composition. The methodology was detailed in the Supplemental Document and was similar to those previously reported by us [24].

### 2.6. Bioinformatics

Barcode-sorted samples were analyzed using the Greengenes V13_8 designed for the taxonomic classification of 16SrRNA sequences. Sequencing reads were clustered into operational taxonomic units (OTUs, 1% distance), described by community metrics, and taxonomically classified within the Qiime 1.8 bioinformatics pipeline. Before creating the phyloseq object for the 16S data, the taxonomic table was evaluated to eliminate species and phyla which were annotated as “unidentified” or lacking identification at a species level with an annotation “empty cell”. This elimination ensured only identifiable species were utilized in the bioinformatic analysis. With regards to total read counts per sample, a cutoff of 500 read counts was utilized as the minimum needed read count.

### 2.7. Statistical Analysis

For the clinical data, statistical analysis was performed using SSPS 24. Fisher exact and Mann–Whitney U tests were applied for statistical analysis; results with *p*-values less than 0.05 were deemed significant.

Alpha and beta diversities, richness, and evenness of the gut microbiome and the relative abundance analysis differences were compared between groups. Non-parametric multivariate distance-based associations between bacterial communities and outcomes were performed using the Adonis function as implemented in the R package vegan version 2.5-2 using (Bray–Curtis) dissimilarity distance metric and its standardized binary form based on presence/absence data instead of abundance. Student *t*-test was used for evaluating continuous variables with the parametric distribution. Non-parametric Spearman correlation and Wilcoxon rank-sum test were used for association with continuous outcome and binary outcomes, respectively. Diversity was analyzed in an unbiased manner using the Shannon diversity index, a measure of abundance taking microbial distribution into account. Richness was also assessed using the observed taxon richness index, reflecting the microbial counts of the bacterial communities in each sample.

Longitudinal analysis was performed using all pairwise multiple comparisons of mean ranks as implemented in the PMCMR plus R package version 1.2.0, employing the Kruskal–Wallis test followed by Bonferroni–Dunn post hoc adjustment. A *p*-value < 0.05 was considered statistically significant for all tests after correcting for multiple comparisons. Correction for multiple tests was performed using the Benjamini–Hochberg adjustment.

Box plots were utilized to show the interquartile range (IQR) as a measure of statistical dispersion and the difference between the groups. The lines extending parallel to the boxes (whiskers) indicated variability outside the upper and lower quartiles, and the central line inside the box represented the median value.

## 3. Results

### 3.1. Demographic and Clinical Characteristics of the Participants

A total of 41 participants were initially enrolled in this study, and among these 36, stool samples were collected. Eight samples were excluded and the reasons for exclusion included unidentified samples and low read counts. For the final analysis, we had a total of twenty-eight patients and among these, eleven received BTFs (all received commercial BTFs and none of the participants received home BTFs) and seventeen received SEF after verification of inclusion and exclusion criteria at the time of the sample collection and completion of quality control analysis. Among the SEF group, ten patients were on polymeric formulas, six patients were on elemental formulas, and one patient was on a protein hydroxylate formula, and all were categorized together as the SEF group due to the smaller sample size, and this method of combining patients on different enteral formulas in one group has been reported by other investigators [9]. A total of twenty-eight patients (BTFs, n = 11; SEF, n = 17) were included in this study.

No significant differences in the demographics were noted between the groups (Table 1). Patients on BTFs had no emesis compared to 41.1% of patients in the control group (*p* = 0.02) (Table 1). There were no significant differences in other clinical characteristics or GI medication usage. Additionally, no significant differences were noted in clinical comorbid conditions (Table 2).

### 3.2. Microbiome Composition

#### 3.2.1. Alpha Diversity

**Figure 1:**
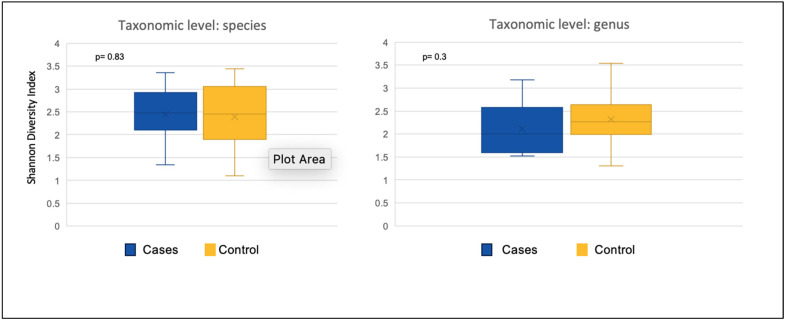
Alpha diversity at the species and genus level between the participants on blenderized tube feeds (cases) and the participants on standard enteral feeds (control group).

There was no significant difference between participants who received BTFs (cases) and participants who received SEF (controls) in the alpha diversity at both species (*p* = 0.83) and genus (*p* = 0.3) levels (Figure 1).

#### 3.2.2. Beta Diversity

**Figure 2:**
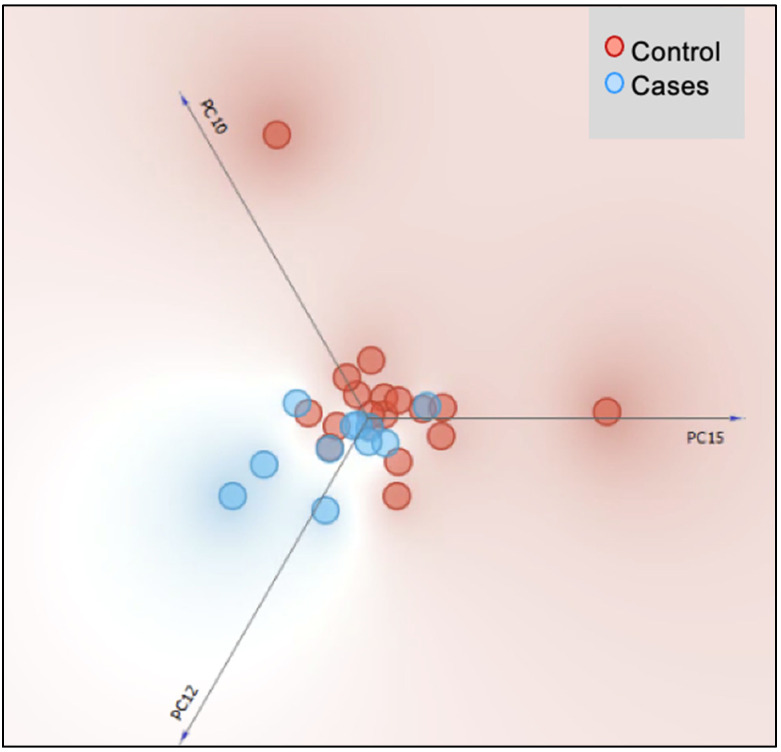
Principal component analysis (PCA) plot of gut bacterial community at the genus level of participants on blenderized tube feeds (cases) and standard enteral formula feeds (control group). There were two outliers in the control group.

The beta diversity based on the principal component analysis plot demonstrated minor and insignificant separation distance (with 82% covariance) between the two groups’ clusters at both genus (Figure 2) and species levels.

#### 3.2.3. Richness and Evenness Indices

**Figure 3:**
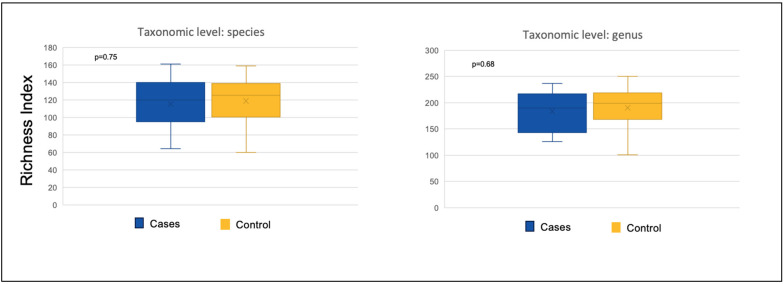
Richness index of bacteria at the species and genus level comparing the participants on blenderized tube feeds (cases) and the participants on standard enteral formula feeds (control group). Y axis notes the number of operational taxonomic units (OTUs).

The richness index between the groups showed no statistically significant difference at species (*p* = 0.75) and genus (*p* = 0.68) levels (Figure 3). Additionally, no significant differences in evenness were found between the groups. Table 3 demonstrated the lack of significance in richness, evenness, and alpha diversity between the two groups at both species and genus levels.

#### 3.2.4. Relative Abundance

The differential abundance analysis noted only a few statistically significant differences across all taxonomic levels (Table 4).

At the species level, the most prevalent and significant differences noted were in the relative abundance of *Bacteroides caccae*, *Enterobacter nickellidurans*, *Pseudoalteromonas piscicida* (Table 4 and Supplemental Figure S1). Supplemental Figures S2 and S3 note the relative abundance at the species level of the most prevalent species (species with a prevalence at 10% or higher were included) and no statistical significance was found between the groups (except for *Bacteroides caccae)*. Notably, the beneficial species, such as *Faecalibacterium prausnitzii* and *Akkermansia muciniphila,* were elevated in the BTF group but this difference did not reach statistical significance (Supplemental Figures S2 and S3). There were nine unique species noted in the SEF group and none in the BTF group (Supplemental Figure S4).

At the genus level, *Aggregatibacter*, *Deinococcus*, and *Exiguobacterium* were elevated in the BTF group, while *Geobacter*, *Thermomonas*, *Clostridium*, *Ramlibacter*, and *Streptococcus* were elevated in the SEF group (Table 4 and Supplemental Figure S5). The most abundant genera (genera with a prevalence at 10% or higher were included) are noted in Supplemental Figures S6 and S7. There were no statistically significant differences between the groups except for *Clostridium*. At the phyla level, the most common abundant phylum was Proteobacteria followed by Firmicutes, Bacteroides, and Actinobacteria (Supplemental Figures S8 and S9). But no significant differences in relative abundance were noted at the phylum level between the groups except for Spirochaetes, which was significantly more abundant in the SEF group (Table 4). In Supplemental Table S1, the mean relative abundance between the BTF and SEF groups, the effect size and fold change are detailed at the species, genus, and phylum levels. Supplemental Table S2 noted the *p*-value differences between the groups at these three taxonomy levels.

We also separated the SEF group (*n* = 17) into a polymeric group (*n* = 10) and non-polymeric group (*n* = 7). The non-polymeric group included participants who received an elemental diet (*n* = 6) and protein hydroxylate (*n* = 1), and we term this group the elemental group (as majority of the patients received elemental formula) in Supplemental Table S3 and Supplemental Figures S10–S15. No significant additional findings were noted with this sub-classification. Supplemental Figure S10 notes the lack of significance in the beta diversity between the three groups. Supplemental Table S3 notes the Shannon diversity index, richness, and Pielou’s evenness index between the BTF, polymeric, and non-polymeric formula groups. No statistical significance was noted between these microbiome parameters. Supplemental Figures S11–S15 detail the relative abundance at different taxonomy levels between the three groups. 

A summary of the study results was presented at the Seventh World Congress of Pediatric Gastroenterology Hepatology and Nutrition, Buenos Aires, Argentina, 4–7 December 2024 [25].

## 4. Discussion

The primary goal of this prospective, observational study was to compare the GI characteristics and gut microbiome between patients on BTFs and standard enteral formulas. We observed a decreased prevalence in vomiting in the BTFs group (0%) compared to 41% in the SEF group. No additional significant differences were noted in other clinical characteristics and comorbidities. No significant differences were noted in alpha and beta diversities, richness, and evenness (at genus and species levels) between the groups. Differential abundance analysis showed only a few significant differences between the groups at all reported taxonomic levels.

Our findings suggested that BTFs may be beneficial for patients with vomiting. A prospective study with 20 pediatric patients showed a decreased prevalence of vomiting and the use of acid suppression in patients who received BTFs [10]. Other studies investigating BTFs also revealed improvement of upper GI symptoms [10,11,12,13,22,26], decrease in loose stools [13], decreased emergency room visits and hospital stays, greater improvement in quality of life and GI symptom scale, and increased caregiver satisfaction ratings [27,28]. Similarly, in an observational study involving gastrostomy-fed children comparing home blenderized diet vs. formula feeds, bowel disturbances were significantly reduced in the home BTF compared to the formula diet (6.3% vs. 70%, *p* = 0.009), respectively, and specifically, total vomiting/retching episodes were less prevalent in BTF (12.5% vs. 50% in formula diet, *p* < 0.001) [28]. The exact reason for improvements in GI symptoms for patients on BTF remains elusive but changes in microbiome are often implicated. Evidence consistently highlights the beneficial effects of dietary fiber on the gut microbiome and human health and blenderized formulas have higher fiber content than SEF [20,21].

Our findings in the bacterial composition analysis did not show a significant difference in the alpha and beta diversity between groups. These findings differed from a recently published study, which showed increased alpha diversity between patients on home BTF (*n* = 5) and SEF (*n* = 6) [21]. Gallager et al. transitioned 17 pediatric patients to home blenderized feeds and described a rise in alpha diversity in stool samples over time [10]. These studies used home-blenderized feeds, whereas our study participants used commercially available blenderized formulas, which could explain the differences observed. Similar to our study findings, McClanahan et al. included 10 children and noted non-significant improvement in the gut microbiome at 2 weeks and 2 months of switching the formula from SEF to a commercial BTF [22].

The most prominent and statistically significant finding at the species level was the increased relative abundance of *Bacteroides caccae* in the BTF group. An overabundance of *Bacteroides caccae* has been associated with the regulation of mucus and decreased intestinal inflammation [29]. At the species level, *Faecalibacterium prausnitzii* was the most abundant in both groups, with a 5% higher abundance in the BTF group. *Faecalibacterium prausnitzii* produces butyrate and bioactive anti-inflammatory molecules [30,31]. This bacterium is considered a bioindicator of intestinal health [32,33]. *Akkermansia muciniphila* relative abundance was also higher in the BTF group, yet this difference did not reach statistical significance. *Akkermansia muciniphila* is known to positively regulate mucin, enhancing the barrier of the gut mucosa [34]. In this study, Proteobacteria, Firmicutes, Bacteroidetes, and Actinobacteria were the most abundant phyla in both groups. Notably, both groups had an increased relative abundance of Proteobacteria. Several studies associate the expansion of this phylum with dysbiosis [35,36,37,38,39]. An increased abundance of members belonging to this phylum has been associated with inflammatory bowel disease, irritable bowel syndrome, metabolic disorders, and lung diseases [38,39]. The mean relative abundance in BTF was lower than in SEF (40.3 vs. 42.4, with an effect size of −0.08, and BTF/SEF fold change was −1.05). Longitudinal research studies with larger sample sizes will help us in confirming or refuting the results of this study.

### Limitations

There are several limitations to this study. This is a single-center exploratory study which limits the possibility of generalizing the findings. All the participants in the BTF group were on commercial blenderized preparations. Future studies should include patients on homemade blenderized feeds. We used semi-structured caregiver interviews and recall bias was possible. The gut microbiome of children <3 years of age is known to be highly dynamic. However, we included patients <3 years old in both groups and there were no significant age differences between the groups. We utilized strict inclusion criteria and standardized stool collection methodology, which helped to decrease cofounders but also led to a small sample and exclusion of many patients. The small sample size (specifically in the BTF group) reduced the power of the study and potentially affected the results. Similarly, medications such as acid suppression, laxatives, erythromycin, and probiotics could alter the microbiome and participants on these medications were not excluded. We also did not evaluate various ingredients (fiber content, macro- and micronutrient composition, etc.) of the diet. A longitudinal design with a larger sample will help us understand the implications of the gut microbiome in patients receiving different enteral feeds, which will enhance ways to possibly modulate the gut microbiome.

## 5. Conclusions

Our cohort consisted of medically complex pediatric and young adult patients receiving enteral tube nutrition and highlighted the high prevalence of GI symptoms and comorbidities. A significant clinical finding of our study was the decreased prevalence of emesis in the BTF group. Despite no differences in diversity, richness, and evenness, and only a few differences in relative abundance, it is important to note that the BTF group was not inferior to the standard and more commonly used SEF group regarding other clinical outcomes and importantly in the gut microbial composition. Longitudinal research studies with larger sample sizes are necessary to better understand the implications of our findings.

## Figures and Tables

**Table 1:** Demographic and clinical characteristics of the study population.

Parameters	Blenderized Tube Feeds (*n* = 11)	Standard Enteral Feeds (*n* = 17)	*p*-Value
Median age (in years)	4.4 (1.5 to 16.5)	2.2 (1.65 to 5.9)	0.14
Sex			0.46
Male	7 (64%)	8 (47%)	
Female	4 (36%)	9 (53%)	
Malnutrition	3 (27.3%)	1 (5.9%)	0.12
Diarrhea	0 (0%)	1 (5.9%)	1.00
Constipation	6 (54.5%)	8 (47.1%)	1.00
Vomiting	0 (0%)	7 (41.1%)	0.02
Oral feeds	5 (45.4%)	4 (25%)	0.58
Acid suppression			0.38
None	5 (45.5%)	4 (23.5%)	
H2RAs	0 (0%)	1 (5.9%)	
PPI	6 (54.5%)	12 (70.6%)	
Use of laxatives	6 (54.5%)	9 (52.9%)	1.00
Use of erythromycin	3 (27.3%)	4 (23.5%)	1.00
Use of probiotics	3 (27.3%)	1 (5.9%)	0.27

H2RAs—H2 receptor antagonists, PPI—proton pump inhibitors.

**Table 2:** Comparison of clinical comorbidities between the two groups.

Parameters	Blenderized Tube Feeds (*n* = 11)	Standard Enteral Feeds (*n* = 17)	*p*-Value
Cerebral palsy	4 (36.4%)	3 (17.6%)	0.38
Autism	2 (18.2%)	1 (5.9%)	0.54
Developmental delay	5 (45.5%)	10 (58.8%)	0.70
Genetic disorders	3 (27.3%)	3 (17.6%)	0.65
Prematurity	3 (27.3%)	5 (29.4%)	1
Oral aversion	6 (54.5%)	7 (41.2%)	0.70
Aspiration	2 (18.2%)	2 (11.8%)	1
GERD	8 (72.7%)	10 (58.8%)	0.68
Food allergies	3 (27.3%)	1 (5.9%)	0.27
CMPI	1 (9.1%)	1 (5.9%)	1
Gastroparesis	2 (18.2%)	2 (11.8%)	1
Epilepsy	4 (36.4%)	4 (23.5%)	0.67
Failure to thrive	5 (45.5%)	3 (17.6%)	0.20
Cardiac disorders	2 (18.2%)	3 (17.6%)	1
Chronic lung disease	4 (36.4%)	7 (41.2%)	1

CMPI—cow’s milk protein intolerance; GERD—gastroesophageal reflux disease.

**Table 3:** Differences in alpha diversity, richness, and evenness between the participants on blenderized tube feeds (BTFs) and the participants on standard enteral formula (SEF) at both species and genus levels.

At Species Level			
Mean Value	Shannon’s Diversity Index	Richness (No. of OTUs)	Pielou’s Evenness Index
BTF (11)	2.44	115	0.52
SEF (17)	2.40	114	0.51
*p*-value BTF vs. SEF	0.83	0.75	0.97
**At Genus Level**			
BTF (11)	2.11	184	0.40
SEF (17)	2.35	183	0.46
BTF vs. SEF *p*-value	0.30	0.68	0.20

**Table 4:** Differential relative abundance (RA) analysis summary across all taxonomic levels between participants on blenderized tube feeds (cases) and on standard enteral formula feeds (control group).

Taxonomic Level	*p*-Value	Elevated in	RA in Cases (%)	RA in Controls (%)	Fold Change (Case/Control)
Species					
*Bacteroides caccae*	0.0222	Cases	1.059	0.213	5.0
*Enterobacter nickellidurans*	0.0486	Cases	0.171	0.028	6.1
*Pseudoalteromonas piscicida*	0.0468	Cases	0.047	0.022	2.2
Genus					
*Aggregatibacter*	0.0032	Cases	0.010	0.002	4.7
*Deinococcus*	0.0041	Cases	0.002	0.000	11.5
*Exiguobacterium*	0.0070	Cases	0.004	0.001	6.2
*Geobacter*	0.0079	Controls	0.000	0.003	Unique to control group
*Thermomonas*	0.0253	Controls	0.000	0.003	−52.4
*Clostridium*	0.0263	Controls	0.001	0.009	−9.9
*Ramlibacter*	0.0294	Controls	0.001	0.006	−4.6
*Streptococcus*	0.0408	Controls	0.084	0.427	−5.1
Phylum					
Spirochaetes	0.0321	Controls	0.007	0.015	−2.1

## Data Availability

The original contributions presented in this study are included in the article/Supplementary Materials. Further inquiries can be directed to the corresponding author.

## Supplementary Materials

The following supporting information can be downloaded at: https://www.mdpi.com/article/10.3390/nu17122018/s1, Figure S1. The most prevalent species with a significant difference between participants on blenderized tube feeds (blended group) and standard enteral formula (control group) were noted, RA-relative abundance. Figure S2. Relative abundance of the most prevalent bacteria species between participants on blenderized tube feeds (cases) and standard enteral formula feeds (control group). Figure S3. Differential relative abundance analysis at species level between participants on blenderized tube feeds (cases) and on standard enteral formula (control group), and no significance was noted between the groups. Figure S4. Venn diagram demonstrating the 9 unique species in the standard enteral formula group (control) and none in the blenderized tube feeds (blended). Figure S5. The most abundant taxonomies with significant differences at the genus level were demonstrated between participants on blenderized tube feeds (blended) and standard enteral formula (control), RA-relative abundance. Figure S6. Relative abundance of the most prevalent genus between participants on blenderized tube feeds (cases) and standard enteral formula (control group). Figure S7. Differential abundance analysis at genus level between participants on blenderized tube feeds (cases) and on standard enteral formula (control group). No statistical significance in relative abundance was noted between the groups. Figure S8. Relative abundance of the most prevalent bacteria phyla between participants on blenderized tube feeds (cases) and standard enteral formula (control group). Figure S9. Differential abundance analysis at phylum level between participants on blenderized tube feeds (cases) and on standard enteral formula (control group). No statistical significance in relative abundance was noted between the groups. Figure S10. Principal Component Analysis (PCA) plot of gut bacterial community at the genus level of participants on blenderized tube feeds (blue), elemental formulas (red) and polymeric formulas (green). Figure S11. Relative abundance of the most prevalent species in all three groups blenderized tube feeds (blended), polymeric, and non-polymeric (elemental) formulas. Figure S12. Relative abundance of the most prevalent species in all three groups blenderized tube feeds (blended), polymeric, and non-polymeric (elemental) formulas. Figure S13. Relative abundance of the most prevalent genera in all three groups blenderized tube feeds (blended), polymeric, and non-polymeric (elemental) formulas. Figure S14. Relative abundance of the most prevalent genera in all three groups blenderized tube feeds (blended), polymeric, and non-polymeric (elemental) formulas. Figure S15. Relative abundance of the most prevalent phyla in all three groups blenderized tube feeds (blended), polymeric, and non-polymeric (elemental) formulas. Table S1. The mean relative abundance (RA) between blenderized tube feeds (blended group) and on standard enteral formula (control group) groups, the effect size and fold change were detailed at species, genus, and phylum levels. Table S2. p Value difference in the mean relative abundance (RA) between blenderized tube feeds (blended group) and on standard enteral formula (control) groups at species, genus, and phylum levels. Table S3. Richness, Shannon Diversity Index (SDI) and Evenness across BTFs, Elemental and Polymeric formulas. No statistical significance noted among any of the indices.
