# Supplementary material for: Clinical and Gut Microbiome Characteristics of Medically Complex Patients Receiving Blenderized Tube Feeds vs. Standard Enteral Feeds
Source: Nutrients. 2025 Jun 17;17(12):2018. doi: 10.3390/nu17122018 (PMC12196097; doi:10.3390/nu17122018)
Supplement: Supplementary file 1 [file nutrients-17-02018-s001.zip › nutrients-3664786-supplementary.pdf]

Supplemental Document - Questionnaire

Data obtained from chart review

1. Age in years and months
2. Gender
3. Height (centimeters)
4. Weight (kilograms)
5. Weight for age Z score
6. Body mass index (BMI) (kg/m2)
7. BMI Z scores

Data obtained from a semi-structured interview using the questionnaire

1. Birth weight
2. Was the patient breast fed as an infant? (Yes/No)
   a. If "yes", duration of breastfeeding
3. Does the patient have a G-tube or a GJ-tube?
   a. If "yes" name ___ and how long has the patient been receiving enteral feeds ______
4. In the past week, who often has then patient had a bowel movement?
   a. Multiple times per day
   b. Daily
   c. Every other day
   d. Two times per week
   e. One time per week
   f. >1 week without bowel movements
5. Does the patient have any vomiting or spitting up episodes? (Yes/No)
   a. If "yes", how many per day _____
6. Does the patient have any gagging? (Yes/No)
   a. If "yes", how many per day _____
7. Does the patient have any retching? (Yes/No)

   a. If “yes”, how many per day ____

8. Does the patient have any diarrhea? (Yes/No)
   a. If “yes”, how many per day ____
9. Does the patient take any acid suppressive medications? (Yes/No)
   a. If “yes” then name ________ , dose_____and duration ______
10. Does the patient take any laxative or stool softener? (Yes/No)
    a. If “yes” then name ______, dose _____ and duration ______
11. Does patient live in a more urban or rural area?
12. Does the patient regularly take any medications (prescription or over the counter)? Please list
13. Does the patient regularly take any probiotics, prebiotics or dietary supplements?
14. Has the patient been treated with antibiotics in the past 2 weeks? (Yes/No)
15. Has the patient been treated with antiviral drugs in the past 2 weeks? (Yes/No)
16. Has the patient been treated with antiparasitic drug in the past 2 weeks? (Yes/No)
17. Does the patient have any history of international travel in the past month?
    a. If yes, name the location
18. Does the patient have a history of abdominal surgery? (Yes/No)
    a. If “Yes” then name the procedure
19. Does the patient have a history of these digestive disorders? (Yes/No) Select all that apply
    a. Irritable bowel syndrome
    b. Celiac disease
    c. Chronic diarrhea
    d. Chronic constipation
    e. Fecal incontinence
    f. Gastroesophageal reflux
    g. Lactose intolerance
    h. Inflammatory bowel disease
    i. Short gut syndrome
    j. Other (please specify) _________
20. What is the type of formula used for feeding?
21. How long has the patient been taking this formula?
22. How many ounces of formula taken by the participant and what is the goal provided by the registered dietitian?

Supplemental Document - Methodology:

DNA Extraction: DNA was extracted using a QIA amp Fast DNA Stool Mini kit (Qiagen GmpH, Hilden, Germany) according to the manufacturer's instructions. Fecal sample swabs were transferred to tubes with 1 ml of InhibitEX lysis Buffer. Swabs were incubated for 1 hour at 75°C and shaken using FastPrep 96 twice for 300 seconds each at 1800 RPM. Equal amounts of 100% ethanol and lysate were mixed in a collecting tube and passed through HiBind DNA Mini Columns (Omega Bio-tek, Georgia, USA) with the resulting DNA pellet eluted using 50 ul Molecular grade water. The quality and purity of the isolated genomic DNA were confirmed by gel electrophoresis and quantitated with the Qubit 2.0 instrument applying the Qubit dsDNA HS Assay (Life Technologies). DNA samples were stored at -20C.

PCR Amplification: Amplifications of the 16S rRNA genes were performed using 16S-515 (5'-GGA CTA CCA GGG TAT CTA ATC CTG- 3') and 16S -804 (5'-(TCC TAC GGG AGG CAG CAG T-3') primers, respectively. The PCR mixture was comprised of Q5 High-Fidelity Master Mix (New England Bioinformatics) at a 1x concentration, along with a double volume of molecular grade water and 0.05 ul/mM each primer. Template DNA (100ng) was added to each 50 ul reaction. Thermo-cycling conditions consisted of an initial denaturation step (3 minutes at 98ºC), followed by 30 cycles of denaturation (10 seconds at 98ºC), annealing (10 seconds at 55ºC for the 16S primers), extension (10 seconds at 72ºC), and a final extension step of 3 minutes at 72ºC. Ten µl of each PCR product were separated using gel electrophoresis on 1.5% agarose gel (containing 7 µg/ml ethidium bromide).

Library Preparation and Sequencing: The amplicon library was cleaned and barcoded, followed by emulsion PCR using Ion Torrent S5 Prime workflow according to the manufacturer's instructions (ThermoFisher Scientific, Waltham, MA, USA). Equal volumes of bacterial 16S rRNA amplicons were pooled, cleaned with AMPure XP beads (Beckman Coulter, CA, USA) to remove unused primers, and then exposed to end repair enzyme for 20 minutes at room temperature. After an additional AMPure clean-up, ligation was performed at 25ºC for 30 minutes using Ion Torrent P1 and a unique barcoded 'A' adaptor per pooled sample after AMPure removal of residual adaptors. All separate barcoded samples were then pooled in equal amounts (10 µl) and sizes selected for the anticipated 16S using Pippin Prep (Sage Bioscience, Beverly, MA, USA). The library was amplified for seven cycles and quantitated on StepOne qPCR instrument ahead of proper dilution to 300 pM going into IonSphere templating reaction on the Ion Chef. Library sequencing was completed on an Ion Torrent S5 sequencer (ThermoFisher Scientific, Waltham, MA, USA).

Supplemental Figure S1 - The most prevalent species with a significant difference between participants on blenderized tube feeds (blended group) and standard enteral formula (control group) were noted, RA-relative abundance.

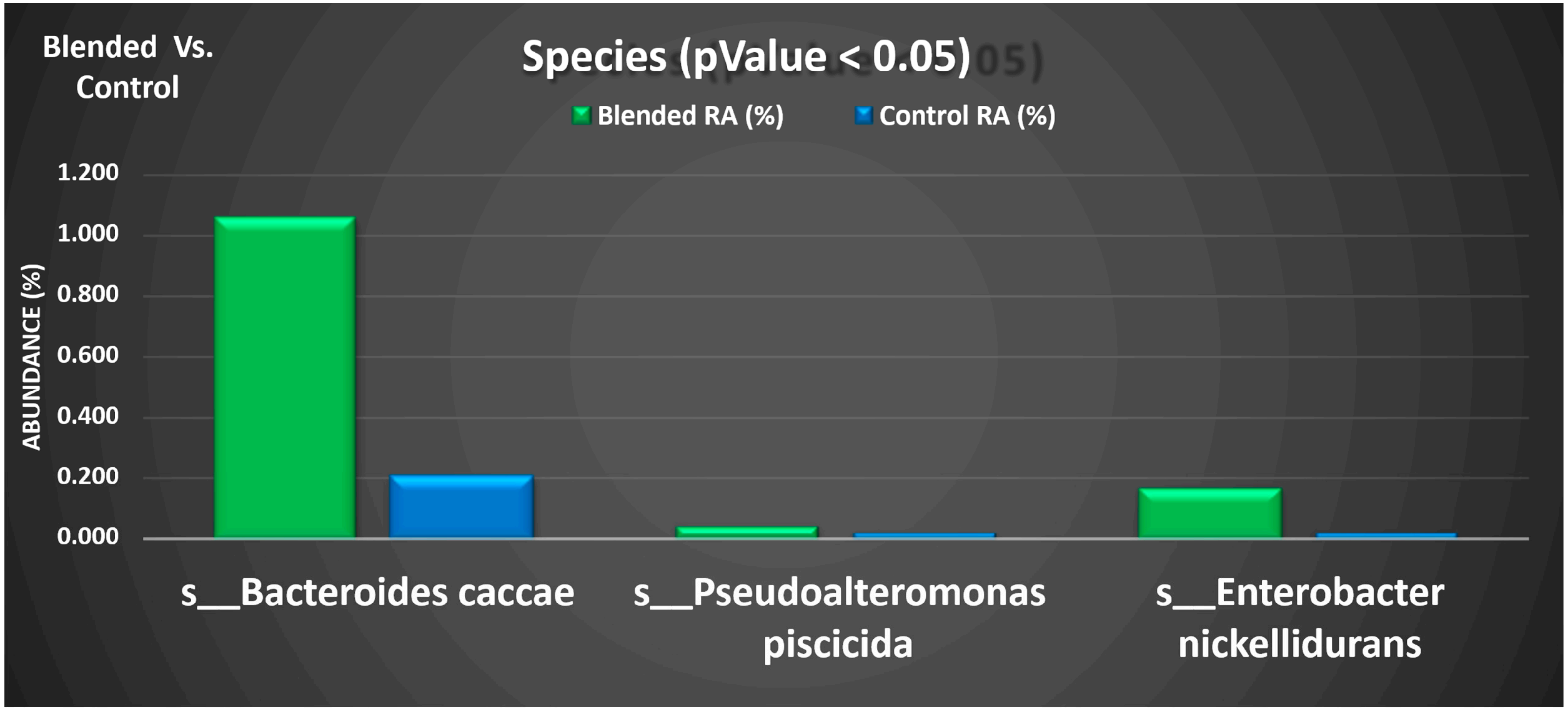

Supplemental Figure S2. Relative abundance of the most prevalent bacteria species between participants on blenderized tube feeds (cases) and standard enteral formula feeds (control group).

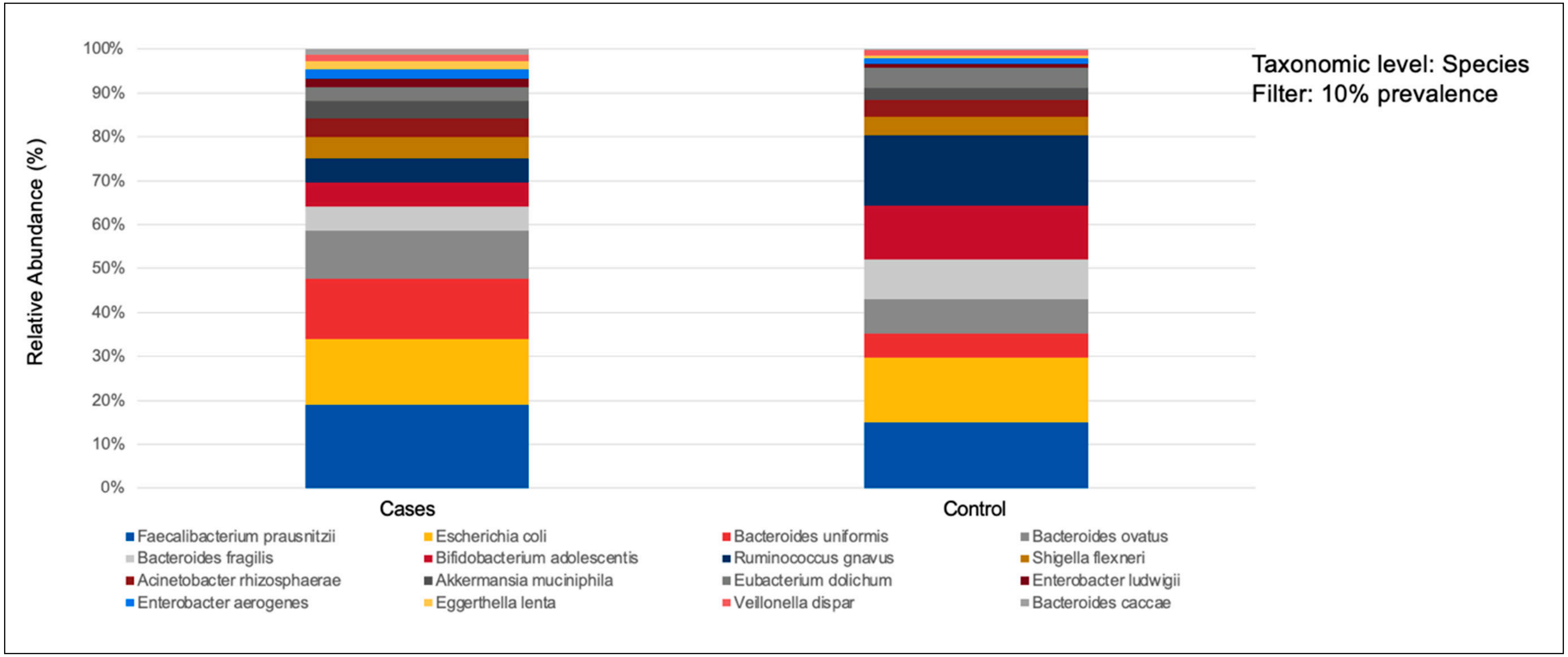

Supplemental Figure S3 - Differential relative abundance analysis at species level between participants on blenderized tube feeds (cases) and on standard enteral formula (control group), and no significance was noted between the groups.

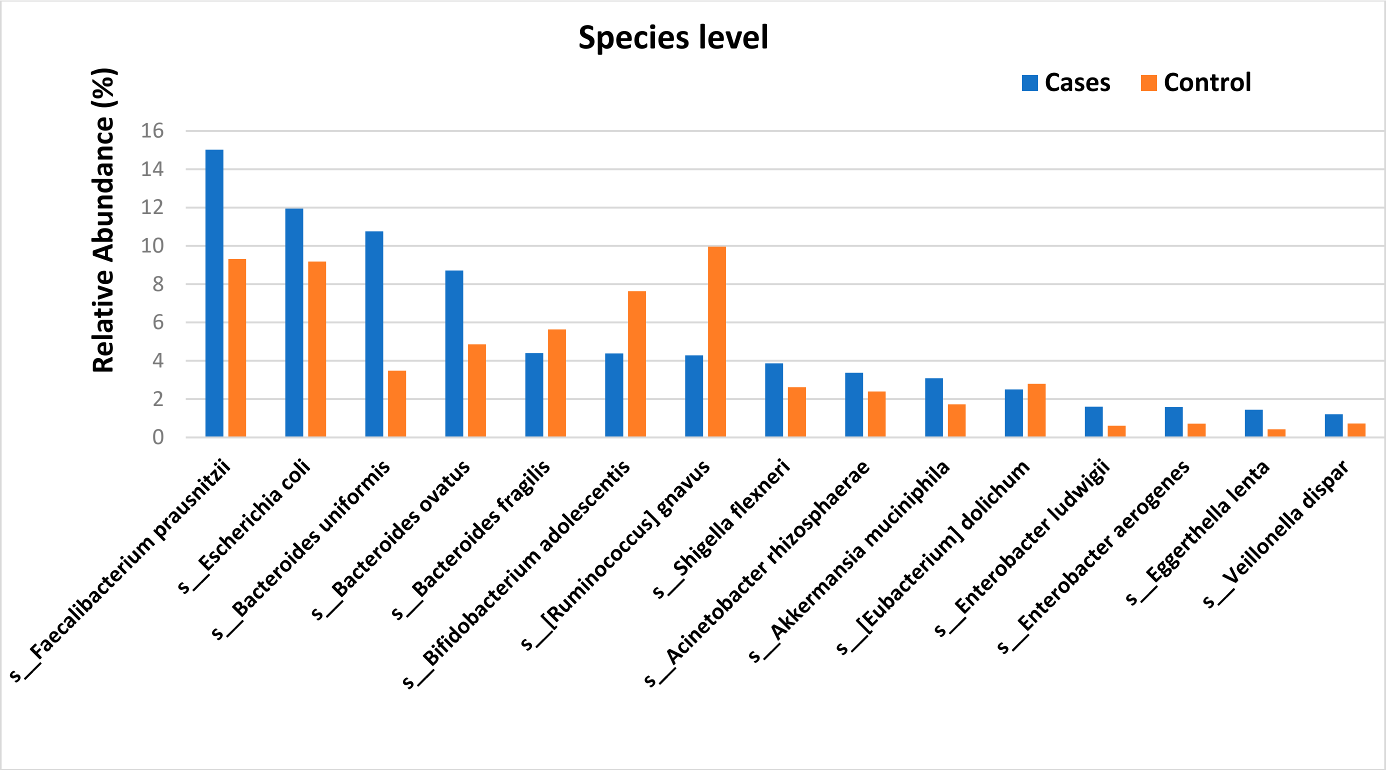

Supplemental Figure S4 – Venn diagram demonstrating the 9 unique species in the standard enteral formula group (control) and none in the blenderized tube feeds (blended).

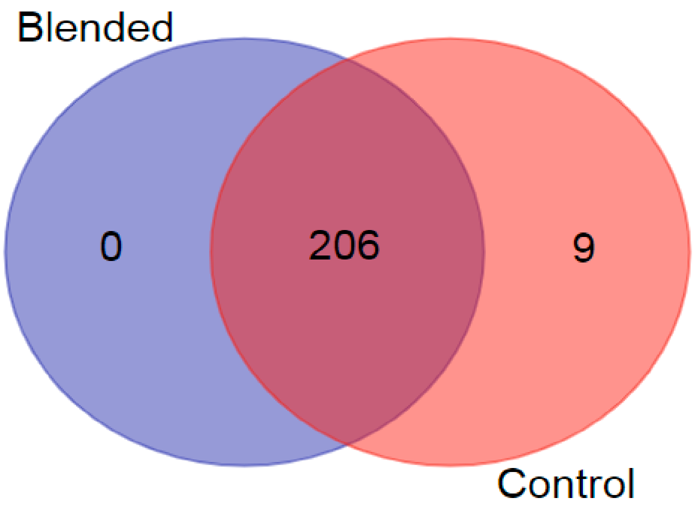

Venn Diagram Results

SPECIES	Blended Vs. Control(FC)	Elevated in	Blended RA (%)	Control RA (%)
s__Streptococcus anginosus	Unique to	Control	0.000	0.014
s__Variovorax paradoxus	Unique to	Control	0.000	0.032
s__Clostridium sphenoides	Unique to	Control	0.000	0.017
s__Roseateles depolymerans	Unique to	Control	0.000	0.062
s__Rhodobacter sphaeroides	Unique to	Control	0.000	0.070
s__Pyramidobacter piscolens	Unique to	Control	0.000	0.168
s__Rhodopseudomonas palustris	Unique to	Control	0.000	0.011
s__Ochrobactrum intermedium	Unique to	Control	0.000	0.008
s__Gluconacetobacter intermedius	Unique to	Control	0.000	0.001

Supplemental Figure S5 - The most abundant taxonomies with significant differences at the genus level were demonstrated between participants on blenderized tube feeds (blended) and standard enteral formula (control), RA-relative abundance.

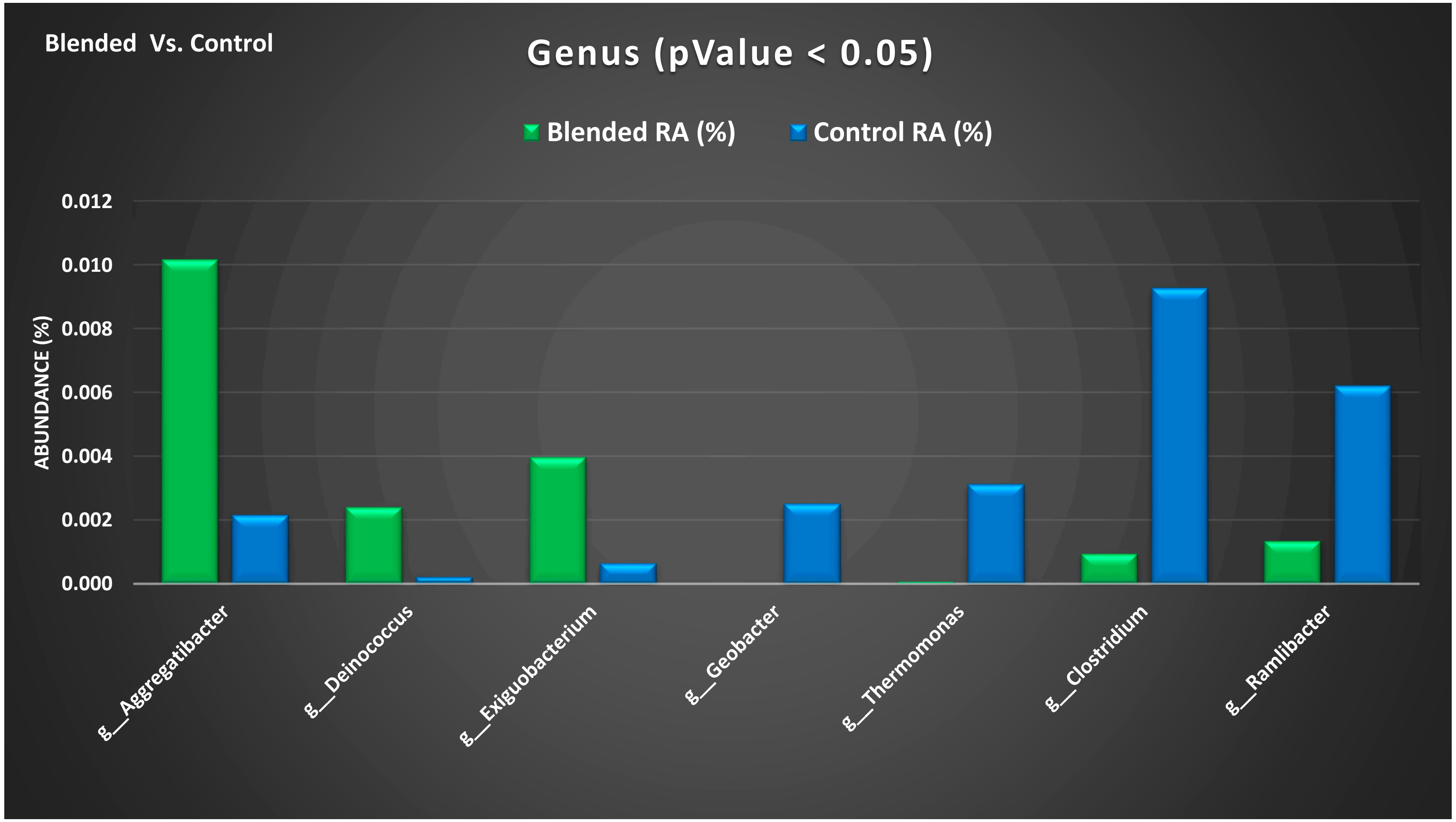

Supplemental Figure S6 - Relative abundance of the most prevalent genus between participants on blenderized tube feeds (cases) and standard enteral formula (control group).

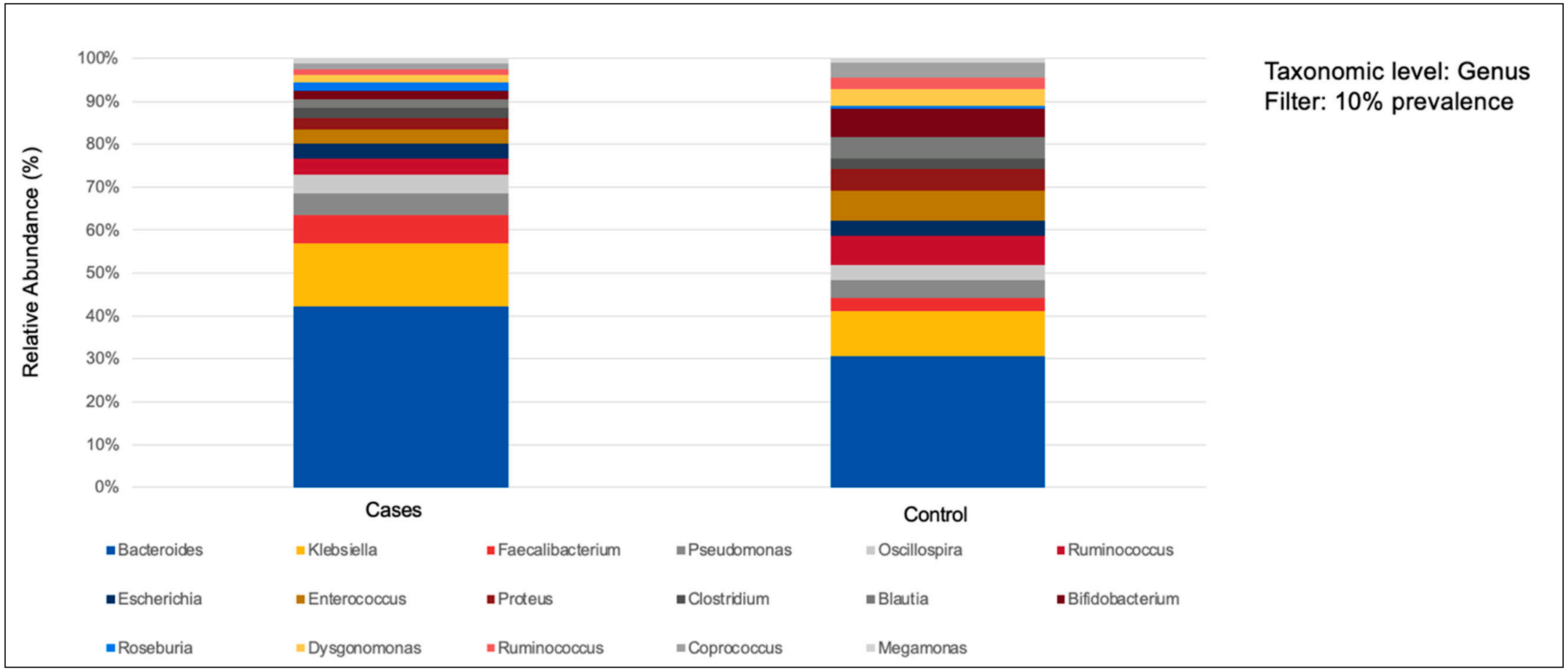

Supplemental Figure S7 - Differential abundance analysis at genus level between participants on blenderized tube feeds (cases) and on standard enteral formula (control group). No statistical significance in relative abundance was noted between the groups.

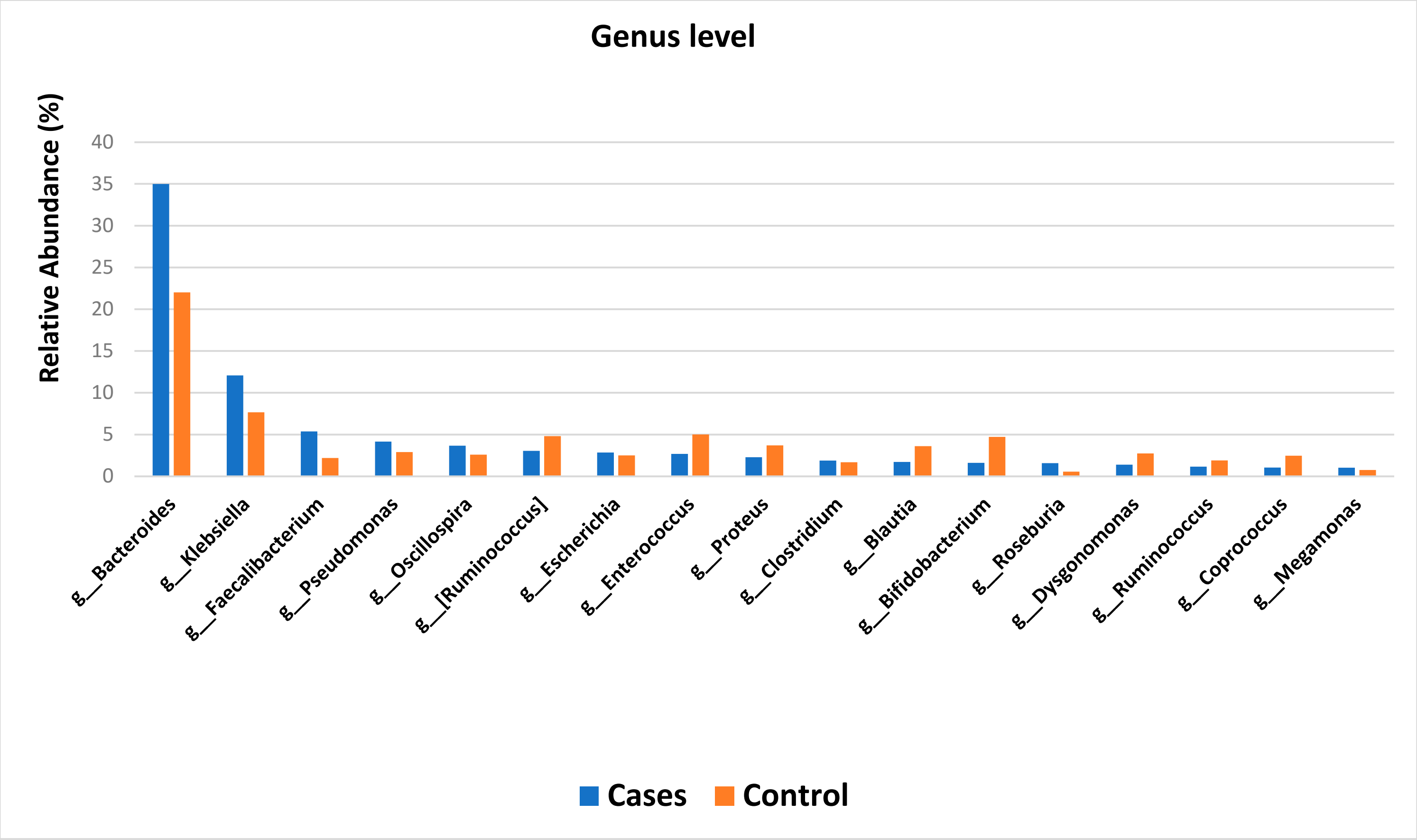

Supplemental Figure S8 - Relative abundance of the most prevalent bacteria phyla between participants on blenderized tube feeds (cases) and standard enteral formula (control group).

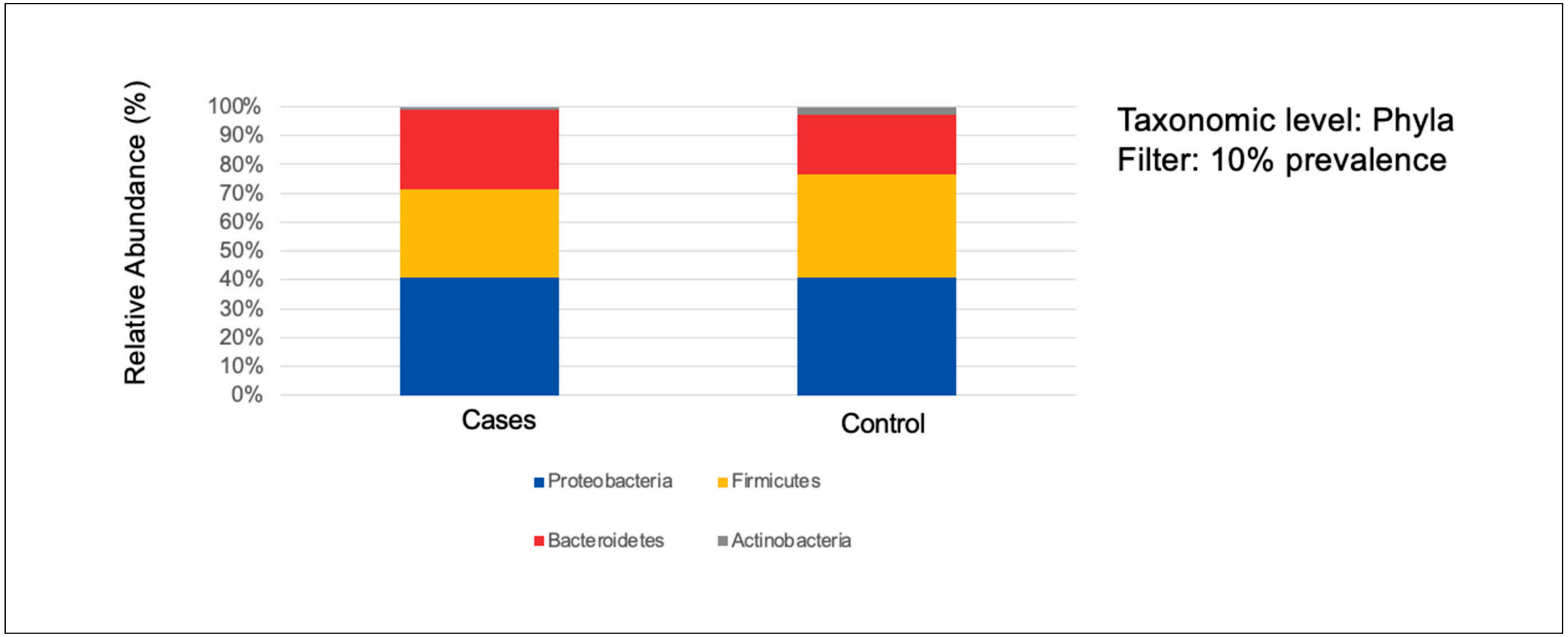

Supplemental Figure S9 - Differential abundance analysis at phylum level between participants on blenderized tube feeds (cases) and on standard enteral formula (control group). No statistical significance in relative abundance was noted between the groups.

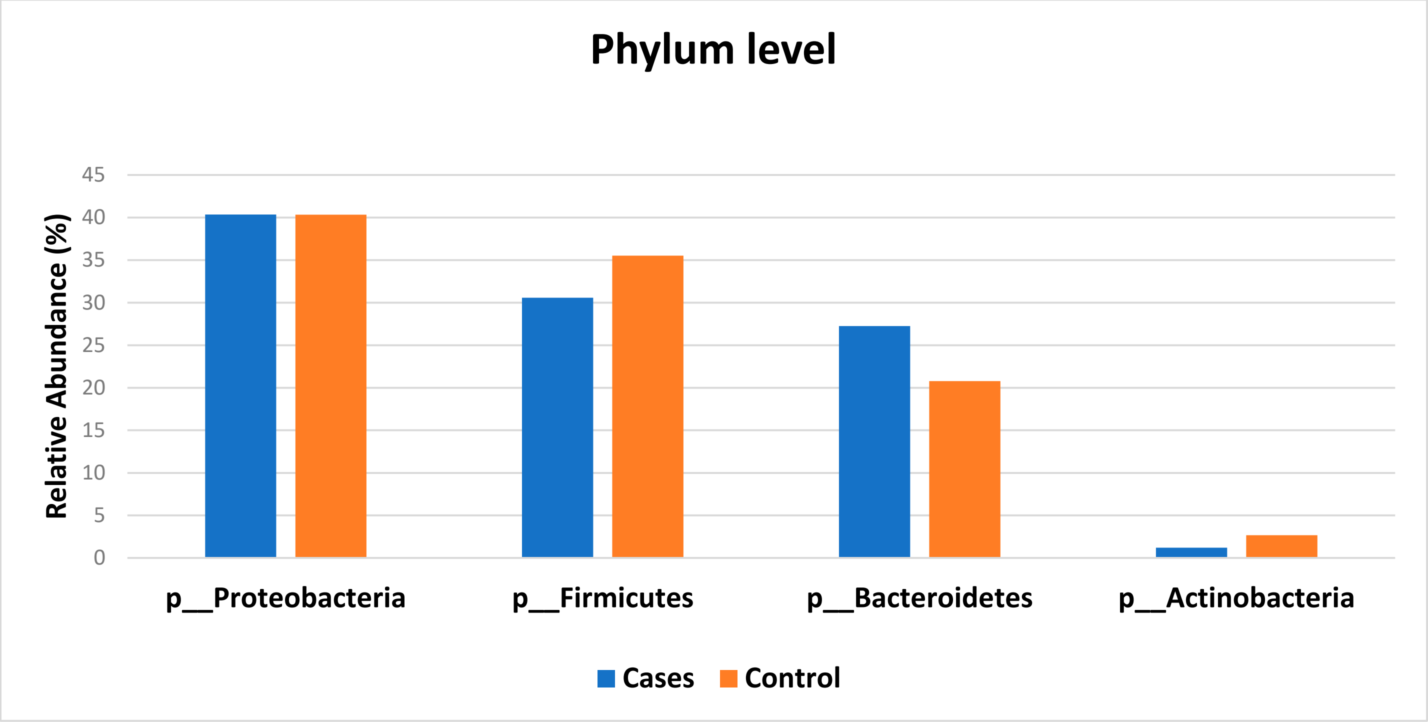

Supplemental Figure S10. Principal Component Analysis (PCA) plot of gut bacterial community at the genus level of participants on blenderized tube feeds (blue), elemental formulas (red) and polymeric formulas (green).

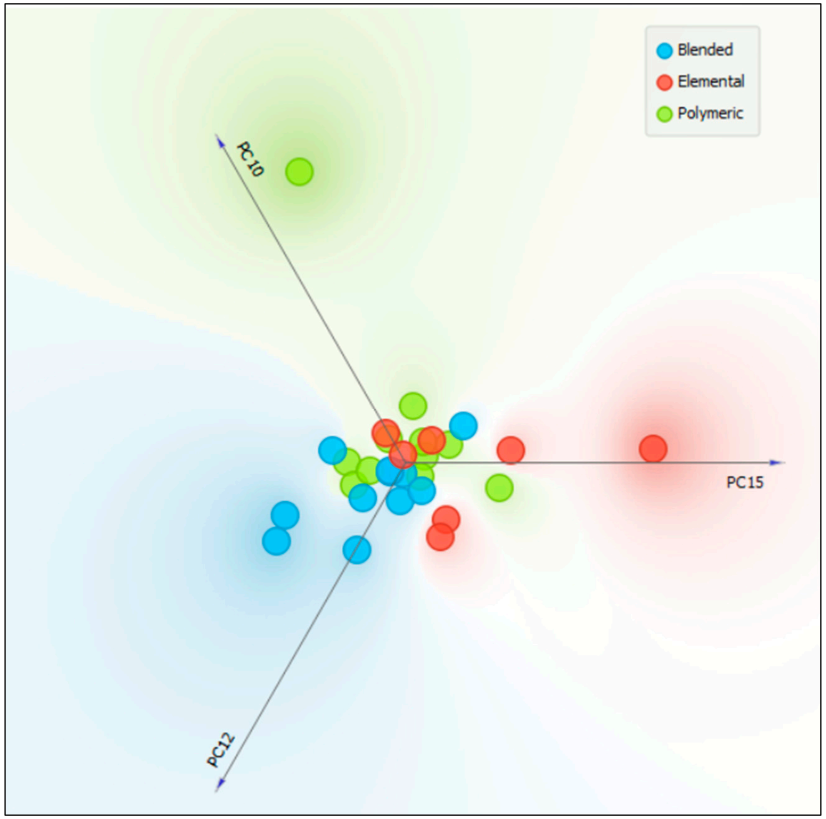

Supplemental Figure S11 – Relative abundance of the most prevalent species in all three groups blenderized tube feeds (blended), polymeric, and non-polymeric (elemental) formulas.

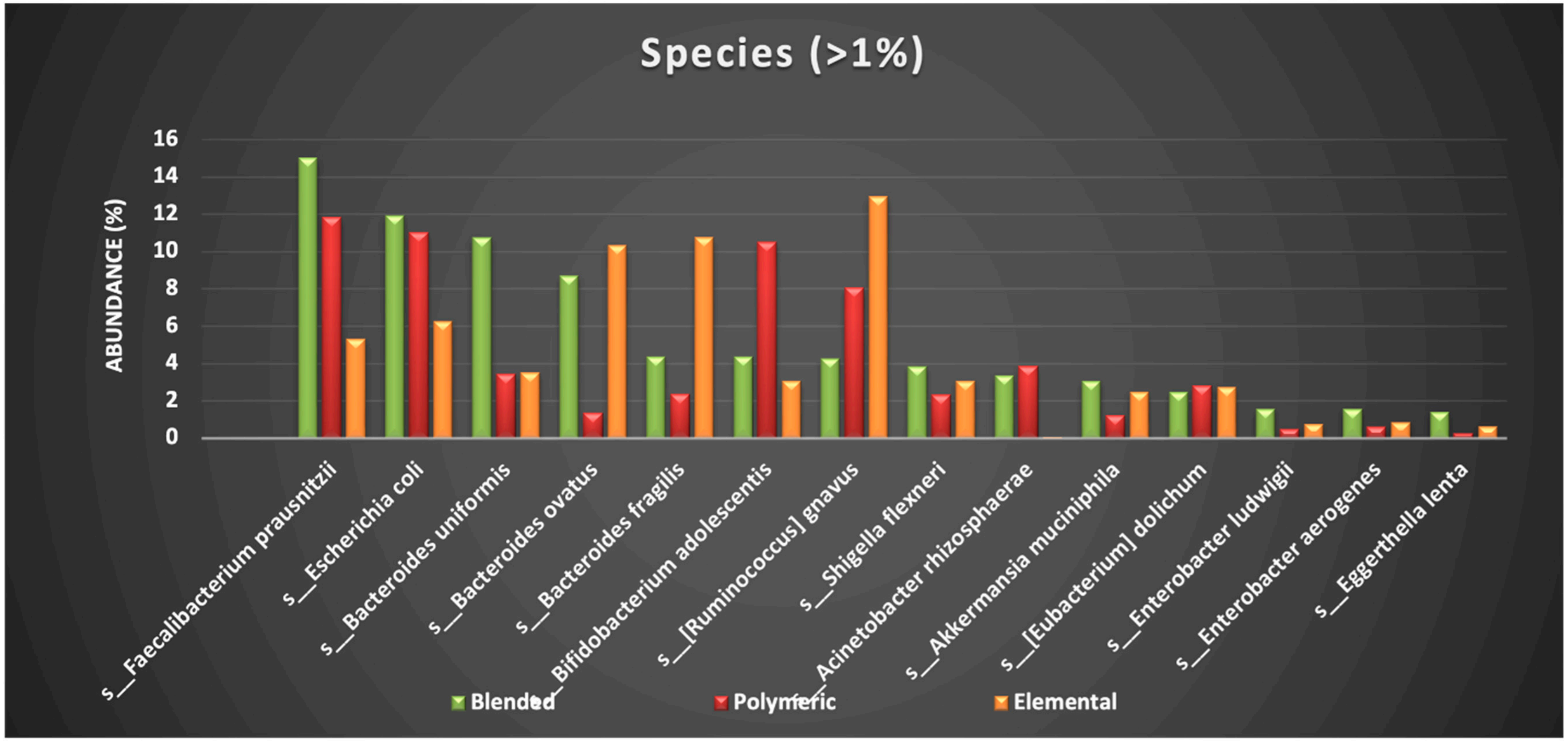

Supplemental Figure S12 – Relative abundance of the most prevalent species in all three groups blenderized tube feeds (blended), polymeric, and non-polymeric (elemental) formulas.

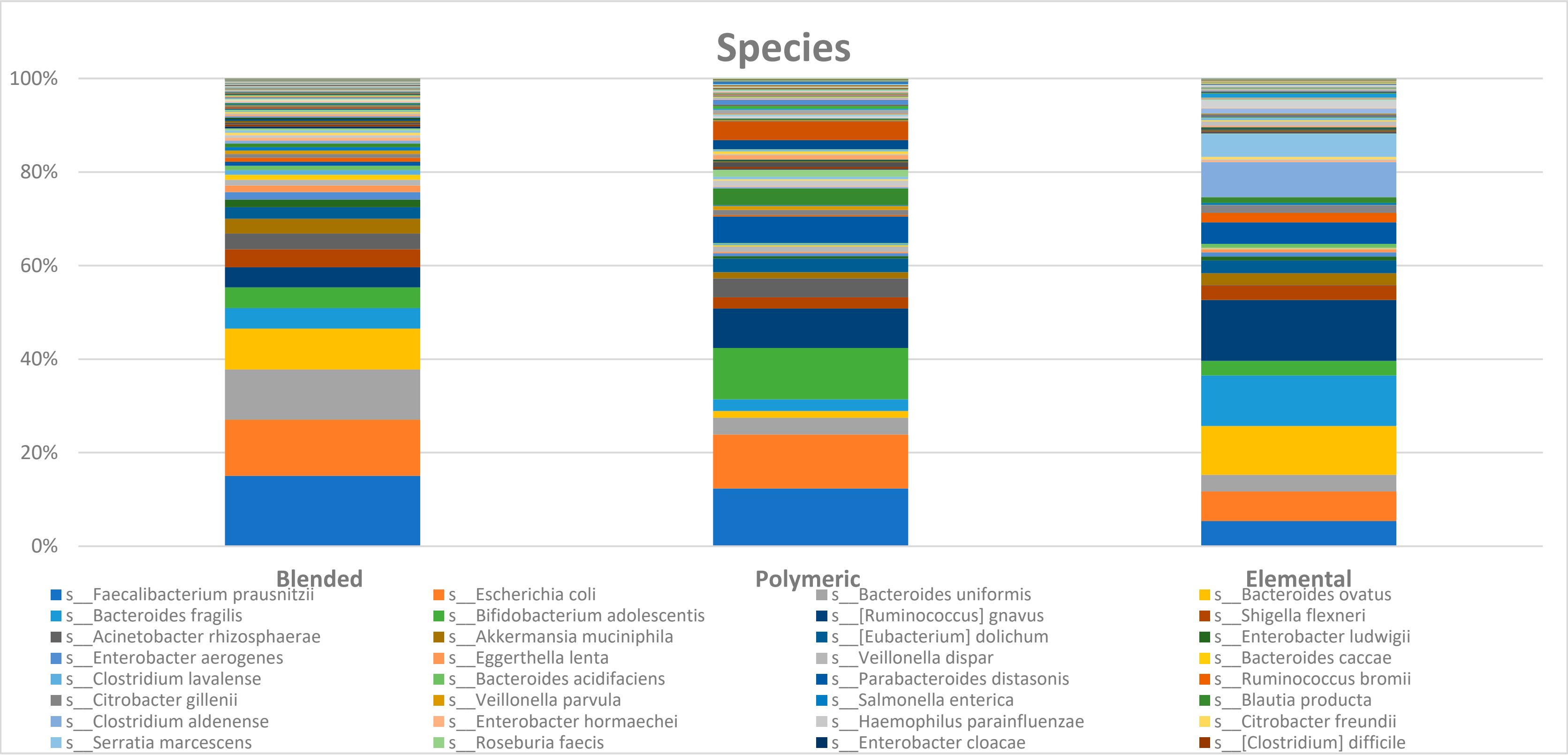

Supplemental Figure S13 – Relative abundance of the most prevalent genera in all three groups blenderized tube feeds (blended), polymeric, and non-polymeric (elemental) formulas.

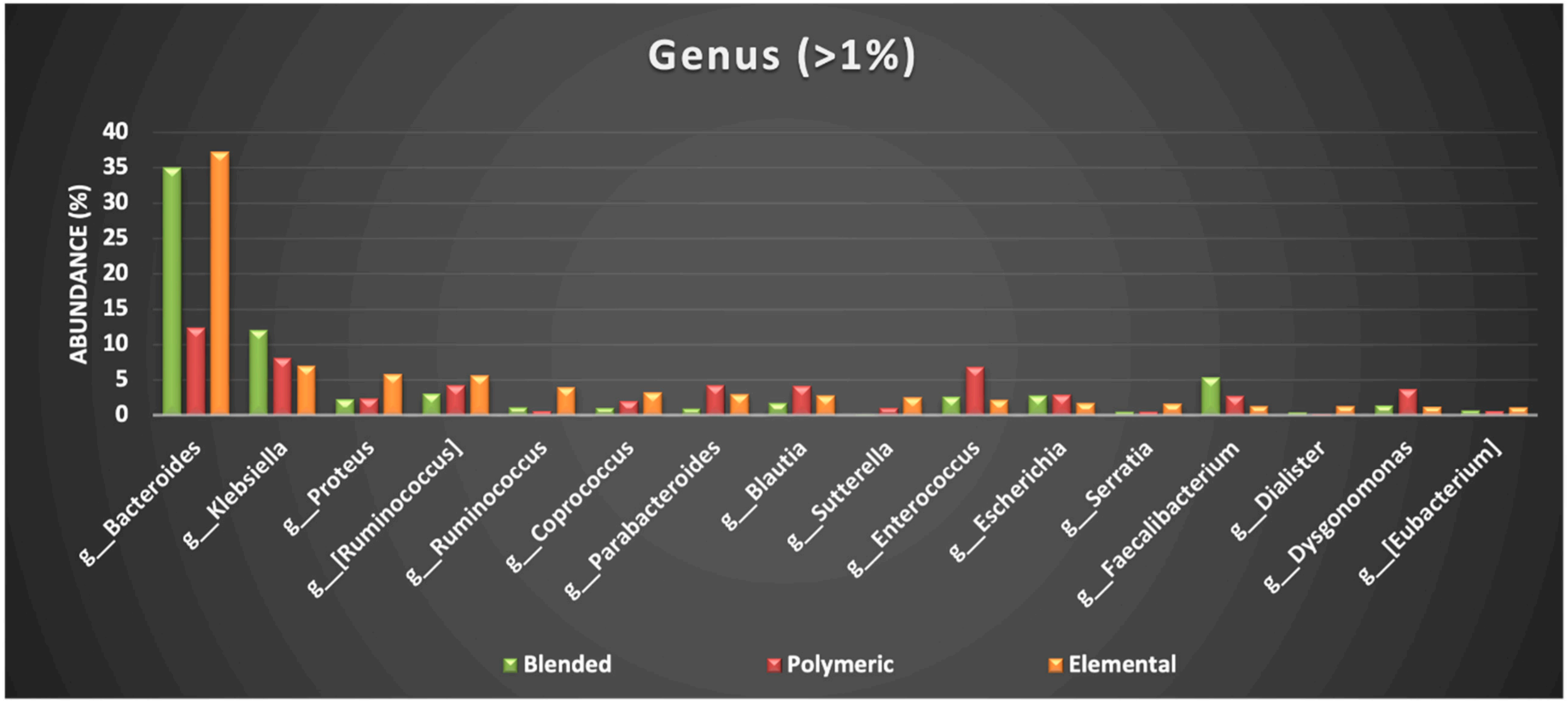

Supplemental Figure S14 – Relative abundance of the most prevalent genera in all three groups blenderized tube feeds (blended), polymeric, and non-polymeric (elemental) formulas.

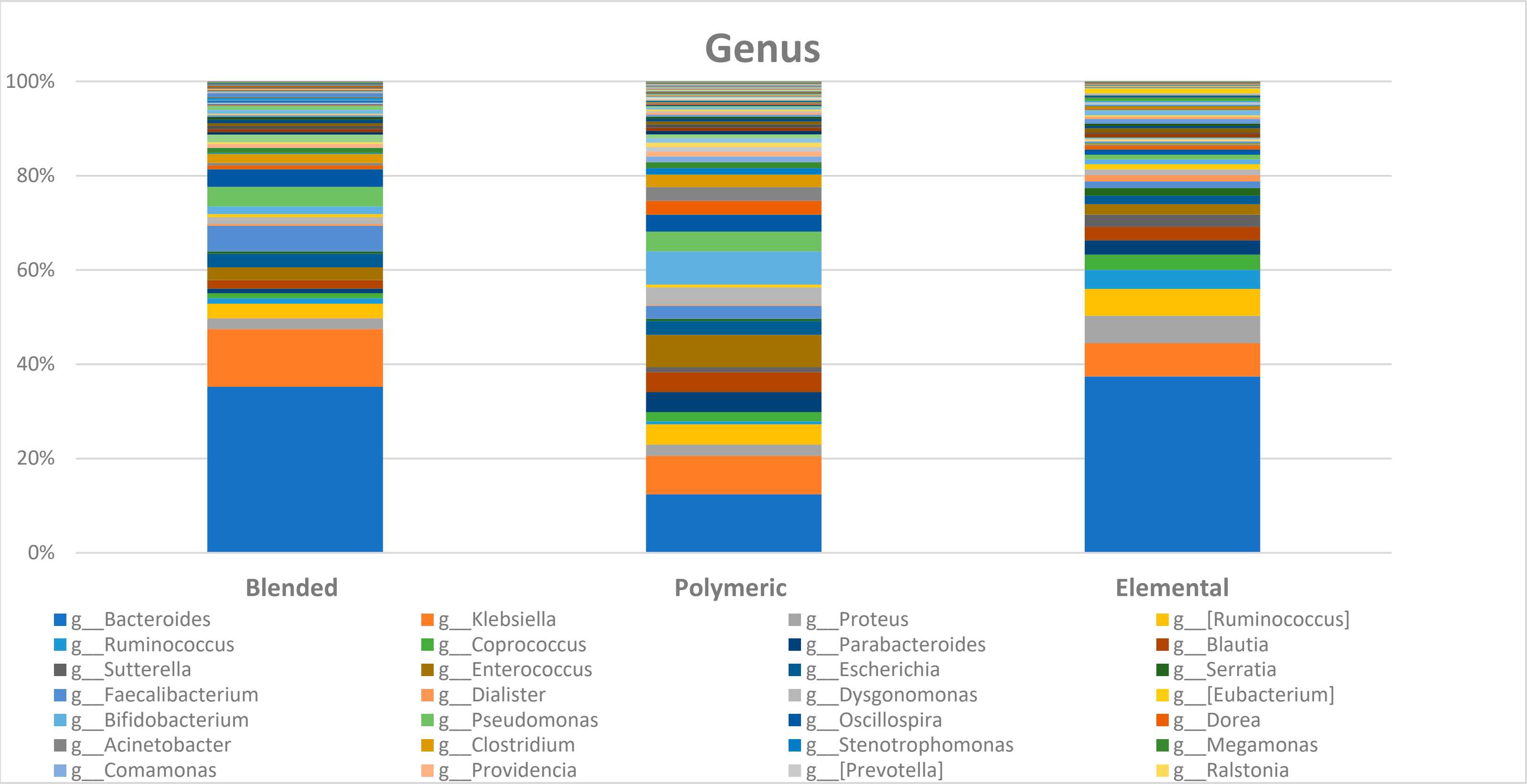

Supplemental Figure S15 – Relative abundance of the most prevalent phyla in all three groups blenderized tube feeds (blended), polymeric, and non-polymeric (elemental) formulas.

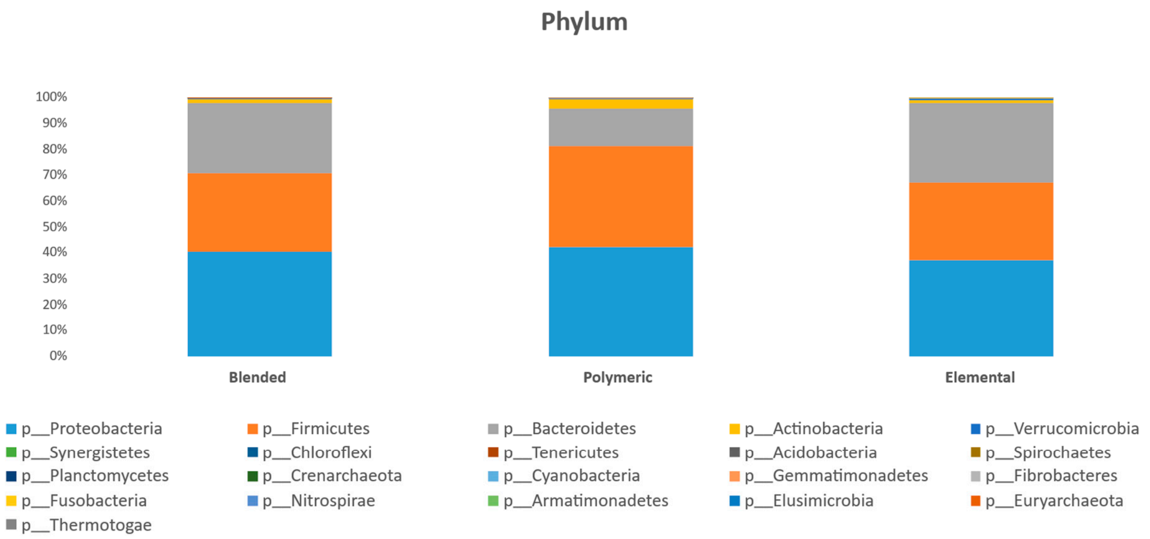

Supplemental Table S1 – The mean relative abundance (RA) between blenderized tube feeds (blended group) and on standard enteral formula (control group) groups, the effect size and fold change were detailed at species, genus, and phylum levels.

Taxonomy Level	Mean RA in Blended	Mean RA in Controls	Standard Deviation	Effect Size	Fold Change
s__Bacteroides ovatus	8.7040875	5.07512638	14.1691785	0.25611655	1.715048424
s__Bifidobacterium adolescentis	4.38700072	8.04050474	15.999635	-0.2283492	0.545612603
s__Faecalibacterium prausnitzii	15.0211315	8.43480444	17.2119704	0.38265968	1.780851185
s__Escherichia coli	11.9408595	9.67883354	13.6338643	0.16591232	1.233708535
s__[Ruminococcus] gnavus	4.28429427	10.1062897	10.679882	-0.5451367	0.423923557
s__Bacteroides uniformis	10.7543832	2.74787947	10.4883325	0.76337241	3.913702646
s__Bacteroides fragilis	4.39968986	5.79430893	10.610088	-0.1314427	0.75931227
s__Clostridium aldenense	0.63055078	3.22286935	9.06540503	-0.2859573	0.195648879
s__Shigella flexneri	3.86069784	2.7789839	4.10235505	0.26368121	1.389248003
s__[Eubacterium] dolichum	2.50623699	2.916467	4.38637036	-0.0935238	0.859340082
s__Acinetobacter rhizosphaerae	3.36950481	2.53367873	8.80885805	0.09488473	1.329886369
s__Parabacteroides distasonis	0.87791945	4.54683894	6.36231847	-0.5766639	0.193083472
s__Clostridium neonatale	0.24602757	2.49354027	7.81896501	-0.2874438	0.098665969
s__Akkermansia muciniphila	3.09554435	1.83073939	5.26920325	0.24003723	1.690871116
s__Bacteroides acidifaciens	0.91789035	0.41118396	1.13327221	0.44711799	2.232310701
s__Blautia producta	0.7366832	2.14310727	2.62765724	-0.5352388	0.343745369
s__Serratia marcescens	0.48556841	2.33321787	6.09038545	-0.3033715	0.208111045
s__Citrobacter gillenii	0.78246137	1.37139818	1.77958131	-0.3309412	0.570557397
s__Enterobacter aerogenes	1.58462439	0.75669344	2.02926338	0.40799581	2.094143157
s__Veillonella dispar	1.21003854	0.77259826	2.08027753	0.21027977	1.56619372
s__Ruminococcus bromii	0.84880306	0.9268738	2.44077967	-0.031986	0.915769829

s__Enterobacter ludwigii	1.59986625	0.59424437	1.74176098	0.57735929	2.692269919
s__Eggerthella lenta	1.44052329	0.44238391	1.75417878	0.56900664	3.256274146
s__Robinsoniella peoriensis	0.08297508	0.66969525	1.65476705	-0.3545636	0.123899753
s__Veillonella parvula	0.7495219	0.54341501	1.63079072	0.12638463	1.379280809
s__Bacteroides caccae	1.05850028	0.21317467	0.99878928	0.90041577	5.049929281
s__[Ruminococcus] torques	0.39285302	0.67813652	1.34124675	-0.2127002	0.579312581
s__Haemophilus parainfluenzae	0.56521491	0.77244255	0.92303044	-0.2245079	0.731724201
s__Clostridium lavalense	0.98398761	0.27209186	1.21294703	0.58691413	3.616380209
s__Collinsella aerofaciens	0.20035451	0.53864614	1.38183825	-0.2448128	0.371959426
s__Roseburia faecis	0.45824148	0.79110561	1.98554693	-0.1676435	0.57924185
s__Acinetobacter johnsonii	0.24661928	1.25481851	2.40572159	-0.4190839	0.196537807
s__Salmonella enterica	0.7407178	0.27948625	0.78471201	0.58777174	2.650283495
s__Citrobacter freundii	0.50878589	0.39585037	0.65273395	0.17301922	1.285298502
s__Bacteroides plebeius	0.29645429	0.42907529	1.31884547	-0.1005584	0.690914373
s__Lactobacillus zeae	0.17532868	0.76635832	1.84390355	-0.3205318	0.228781598
s__Enterobacter hormaechei	0.5963174	0.26205308	0.71243197	0.4691877	2.275559565
s__Raoultella ornithinolytica	0.23487012	0.28864922	0.5450622	-0.098666	0.813687005
s__Bifidobacterium bifidum	0.12497377	0.48092832	1.10128904	-0.3232163	0.259859452
s__Clostridium aerotolerans	0.00217837	1.2382325	3.95143523	-0.3128114	0.001759258
s__Stenotrophomonas acidaminiphila	0.30085431	0.66274799	1.11446792	-0.3247233	0.453949784
s__Erwinia dispersa	0.31364184	0.28399369	0.32245644	0.09194468	1.104397233
s__Prevotella copri	0.2832962	0.22542499	0.13799636	0.41936762	1.256720446
s__Pseudomonas fragi	0.2616689	0.36680086	0.3279994	-0.3205249	0.713381361
s__Klebsiella pneumoniae	0.37366183	0.20650173	0.44590385	0.37487923	1.809485183
s__Enterobacter cloacae	0.43520556	0.1797951	0.47423674	0.53857164	2.42056404
s__Enterobacter cancerogenus	0.36596304	0.1677871	0.43036591	0.46048243	2.181115456
s__Dorea formicigenerans	0.12726838	0.36093044	0.38884803	-0.6009085	0.352611919
s__[Clostridium] difficile	0.40394275	0.31125618	0.74378304	0.12461506	1.297782261
s__Clostridium citroniae	0.12346485	0.43904244	0.87208832	-0.3618643	0.281213928
s__Trabulsiella farmeri	0.25780211	0.15201997	0.26555167	0.39834862	1.6958437

s__Clostridium tertium	0.2087624	0.14550092	0.46813422	0.13513534	1.434784036
s__Citrobacter youngae	0.15085464	0.17782884	0.3127697	-0.086243	0.848313702
s__Bacillus halodurans	0.07901493	0.32269119	0.43478061	-0.560458	0.244862359
s__[Eubacterium] biforme	0.0274575	0.22821399	0.64033779	-0.3135165	0.120314725
s__Staphylococcus epidermidis	0.22282983	0.17737608	0.46566933	0.09760952	1.256256421
s__Pseudoxanthomonas indica	0.1545659	0.18113248	0.16158945	-0.1644079	0.85333067
s__Clostridium saccharolyticum	0.00710904	0.21606572	0.49794988	-0.419634	0.032902222
s__Enterobacter amnigenus	0.18791611	0.1029563	0.24097763	0.35256305	1.82520261
s__Collinsella stercoris	0.05219388	0.15367736	0.28291731	-0.3587037	0.339632852
s__Klebsiella oxytoca	0.30447777	0.06537159	0.3676353	0.65038963	4.657646902
s__Bifidobacterium pseudolongum	0.06808668	0.07768281	0.15064252	-0.0637014	0.876470327
s__Stenotrophomonas geniculata	0.070218	0.16863205	0.39593266	-0.2485626	0.416397693
s__Aquamonas haywardensis	0.11287128	0.13025559	0.21243949	-0.0818318	0.866536895
s__Janthinobacterium lividum	0.09256073	0.08242055	0.15837275	0.06402732	1.123029792
s__Pseudomonas nitroreducens	0.07207461	0.12419718	0.26841448	-0.1941869	0.580324045
s__Cronobacter sakazakii	0.11154943	0.09625107	0.11013233	0.13890893	1.158942285
s__Enterobacter cowanii	0.05891546	0.10784816	0.1416677	-0.3454048	0.546281524
s__Tatumella punctata	0.12511376	0.06252224	0.16790693	0.3727751	2.001108133
s__Pseudomonas alcaligenes	0.13121583	0.12523937	0.30281006	0.01973666	1.047720296
s__Desulfosporosinus meridiei	0.06892858	0.10986572	0.14138316	-0.2895475	0.627389332
s__Enterococcus casseliflavus	0.03956394	0.10269382	0.11921628	-0.5295407	0.385261164
s__Morganella morganii	0.07525124	0.08783666	0.11103814	-0.1133432	0.856717923
s__Acinetobacter radioresistens	0.07529466	0.0506385	0.0631804	0.39025018	1.486905483
s__Bifidobacterium longum	0.04822671	0.0355829	0.1124707	0.1124187	1.355333892
s__Acinetobacter schindleri	0.05073302	0.18557432	0.32850674	-0.4104674	0.273383825
s__Pseudomonas stutzeri	0.06419836	0.09737011	0.15512967	-0.2138324	0.659323055
s__Shigella sonnei	0.07840282	0.06178604	0.08869447	0.18734862	1.268940812
s__Enterobacter nickellidurans	0.17109579	0.02846817	0.19435028	0.7287235	6.106121605
s__Anoxybacillus kestanbolensis	0.08097681	0.0784696	0.12693305	0.01975219	1.031951307
s__Clostridium bartlettii	0.0065557	0.21071696	0.65661852	-0.3109283	0.031111421

s__Dictyostelium citrinum	0.04145101	0.0605909	0.0541126	-0.3537048	0.684112841
s__Blautia obeum	0.03863701	0.0616292	0.08500205	-0.2704897	0.626927116
s__Rhodobacter sphaeroides	0	0.0740778	0.23798961	-0.3112649	0
s__Bacteroides eggerthii	0.06527057	0.05001552	0.07634016	0.19982996	1.305006342
s__Clostridium hathewayi	0.00788065	0.02426627	0.03732332	-0.4390183	0.324757357
s__Clostridium perfringens	0.10171608	0.04187397	0.16337325	0.36629079	2.429100668
s__Lysinibacillus boronitolerans	0.04971467	0.07121741	0.11058227	-0.1944502	0.698069025
s__Plesiomonas shigelloides	0.0533589	0.04814147	0.06183811	0.08437243	1.108377089
s__Pseudomonas veronii	0.07497653	0.0604478	0.1436655	0.10112888	1.240351709
s__Lactonifactor longoviformis	0.11106666	0.0106052	0.15441275	0.65060337	10.47284619
s__Pseudomonas viridiflava	0.05962381	0.1150664	0.27977627	-0.1981676	0.518168697
s__Pantoea agglomerans	0.05021852	0.04222451	0.05934571	0.1347025	1.189321699
s__Histophilus somni	0.03589165	0.04632104	0.05436412	-0.1918433	0.774845485
s__Streptococcus luteciae	0.03600078	0.0653662	0.11814463	-0.2485548	0.550755323
s__Serratia rubidaea	0.02644723	0.05307506	0.08590804	-0.3099573	0.49829866
s__Shigella boydii	0.04280035	0.03233105	0.04554339	0.22987532	1.323815763
s__Pseudoalteromonas piscicida	0.04695182	0.02290963	0.03365254	0.71442432	2.249436262
s__Campylobacter ureolyticus	0.21550573	0.00194974	0.42286252	0.50502464	110.5307315
s__Clostridium clostridioforme	0.03328928	0.03320032	0.05140251	0.00173061	1.002679426
s__Bacillus horneckiae	0.09061232	0.00634558	0.18299757	0.46048009	14.27959553
s__Acinetobacter guillouiae	0.00821546	0.08675944	0.18314676	-0.4288582	0.094692417
s__Staphylococcus aureus	0.03499167	0.0487529	0.09138667	-0.1505824	0.717735252
s__Prevotella nigrescens	0.03950139	0.01566445	0.02522396	0.94501153	2.521721914
s__Bacillus thermoamylovorans	0.13908823	0.00431641	0.28592415	0.47135514	32.22315039
s__Pseudomonas umsongensis	0.04364281	0.03141313	0.05175496	0.23629958	1.389317266
s__Alcaligenes faecalis	0.00435269	0.04150935	0.11755416	-0.3160812	0.104860436
s__Aeromonas sobria	0.03765585	0.02784597	0.0443307	0.2212885	1.352290627
s__Pyramidobacter piscolens	0	0.17767625	0.57082013	-0.3112649	0
s__Halorhodospira halophila	0.0244203	0.02820338	0.04613401	-0.082002	0.865864239
s__Coprobacillus cateniformis	0.0294434	0.04386406	0.08941186	-0.1612834	0.671242184

s__Bulleidia moorei	0.00679344	0.02458924	0.04371642	-0.4070738	0.276276867
s__Dysgonomonas gadei	0.03597096	0.02030952	0.07998545	0.1958036	1.771137764
s__Brevundimonas diminuta	0.00706363	0.10121419	0.25624556	-0.3674232	0.06978892
s__Raoultella terrigena	0.02009021	0.02166357	0.02487294	-0.0632559	0.927373052
s__Lactobacillus plantarum	0.01783347	0.0330298	0.04292363	-0.3540318	0.539920612
s__Alkalimonas amylolytica	0.01484952	0.02751741	0.03185202	-0.3977108	0.539640824
s__Clostridium difficile	0.03981037	0.02338417	0.05336457	0.30781113	1.702450021
s__Serratia ureilytica	0.02815245	0.01666157	0.02835413	0.40526307	1.689663806
s__Listeria weihenstephanensis	0.00966139	0.04561008	0.11327387	-0.3173609	0.211825689
s__Aeromonas hydrophila	0.02898423	0.0170085	0.02630747	0.45522146	1.704102252
s__Enterococcus haemoperoxidus	0.01831263	0.02481612	0.04132425	-0.1573773	0.737932632
s__Weissella viridescens	0.01584416	0.02542985	0.02978407	-0.3218397	0.623053427
s__Roseateles depolymerans	0	0.06564056	0.19417242	-0.338053	0
s__Escherichia hermannii	0.02118473	0.01987986	0.03270361	0.03990011	1.065638181
s__Cedecea neteri	0.03383441	0.0090497	0.04580289	0.54111668	3.738733968
s__Brenneria nigrifluens	0.02144674	0.01076582	0.0236818	0.45101774	1.992112887
s__Candidatus Regiella insecticola	0.0261983	0.018533	0.03481087	0.22019846	1.413602704
s__Bifidobacterium breve	0.01652324	0.00673312	0.03518691	0.27823177	2.454023359
s__Desulfovibrio oxamicus	0.00055183	0.03110477	0.06807571	-0.4488082	0.017741129
s__Bradyrhizobium elkanii	0.0135419	0.01763487	0.01982111	-0.2064952	0.767904979
s__Ruminococcus flavefaciens	0.02014567	0.01708439	0.01922248	0.15925503	1.179185617
s__Alistipes indistinctus	0.00455989	0.01870257	0.04488609	-0.3150792	0.243811159
s__Bacillus flexus	0.01701702	0.02569426	0.03293074	-0.2635	0.662288481
s__Pseudomonas pseudoalcaligenes	0.01873964	0.02221611	0.04747333	-0.07323	0.843515829
s__Leclercia adecarboxylata	0.02075656	0.0132004	0.03220633	0.23461719	1.572419029
s__Clostridium spiroforme	0.00730499	0.02743161	0.03661405	-0.5496966	0.266298129
s__Agrobacterium sullae	0.01738877	0.01941251	0.02236045	-0.0905052	0.895750845
s__Bacillus cereus	0.0095674	0.02142125	0.0262911	-0.4508695	0.446631071
s__Pseudomonas citronellolis	0.01638063	0.02029996	0.02860356	-0.1370226	0.80692902
s__Clostridium methylpentosum	0.04693629	0.00591813	0.08807647	0.46571073	7.93092918

s__Cronobacter dublinensis	0.00711798	0.03030759	0.0901409	-0.2572597	0.234857807
s__Planococcus maitriensis	0.01230527	0.02253394	0.0376051	-0.2720022	0.546077208
s__Schneideria nysicola	0.02051534	0.01162345	0.02028345	0.43838158	1.764996092
s__Clostridium sartagoforme	0.0132838	0.0138324	0.02973217	-0.0184514	0.960339578
s__Acinetobacter lwoffii	0.01178574	0.0205528	0.02611894	-0.3356593	0.573437002
s__Bacillus muralis	0.03097225	0.00065316	0.05987158	0.50640207	47.41913446
s__Pseudomonas mendocina	0.01137074	0.01540565	0.04490521	-0.0898538	0.738089346
s__[Clostridium] sordellii	0.0022064	0.03899322	0.11229623	-0.3275873	0.056584139
s__Pseudidiomarina homiensis	0.01334358	0.01047432	0.01447385	0.19823751	1.273932827
s__Stenotrophomonas maltophilia	0.01043113	0.01187387	0.03109855	-0.0463926	0.878494315
s__Variovorax paradoxus	0	0.03409406	0.08884457	-0.3837495	0
s__Haloferula harenae	0.01697543	0.00651815	0.03762536	0.27793169	2.604332273
s__Clostridium hiranonis	0.00835005	0.02656779	0.05420607	-0.3360831	0.314292125
s__Prevotella stercorea	0.01605033	0.00622411	0.02054136	0.47836262	2.578734392
s__Profftia tarda	0.00893598	0.00970934	0.02013574	-0.0384075	0.920348547
s__Asticcacaulis biprosthecium	0.00256097	0.00425261	0.00872231	-0.1939441	0.602210968
s__Brenneria quercina	0.00291335	0.0132469	0.03129258	-0.3302236	0.219927166
s__Neisseria subflava	0.03002007	0.00911687	0.04360189	0.47941045	3.292804918
s__Aeromonas caviae	0.0177215	0.00745831	0.01889154	0.54326925	2.376074951
s__Staphylococcus equorum	0.00881915	0.01701636	0.02165986	-0.3784518	0.518274588
s__Bifidobacterium animalis	0.00377006	0.01153636	0.0165631	-0.4688913	0.326798316
s__Rahnella aquatilis	0.00613951	0.01001804	0.01163122	-0.3334582	0.612845808
s__[Clostridium] irregulare	0.01990768	0.00734217	0.02328943	0.5395371	2.711416082
s__Shewanella hanedai	0.01220854	0.00678711	0.01920015	0.28236383	1.798782644
s__Rothia mucilaginosa	0.00992159	0.00565621	0.01484061	0.28741245	1.754104402
s__human gut metagenome	0.02107426	0.00751196	0.0428116	0.3167904	2.805428931
s__Erwinia soli	0.01179742	0.00547705	0.01235723	0.51147149	2.153973757
s__Clostridium celatum	0.00956435	0.01339338	0.02355643	-0.1625471	0.714110342
s__Bacillus endophyticus	0.00556152	0.01814176	0.02782399	-0.4521367	0.306558698
s__Enterobacter radicincitans	0.01792204	0.00837799	0.03006157	0.31748353	2.139181821

s__Shewanella benthica	0.01480697	0.00653828	0.01606531	0.5146921	2.264658278
s__Stenotrophomonas rhizophila	0.0028481	0.01610933	0.02710155	-0.4893165	0.176797986
s__Brenneria alni	0.01288794	0.00630852	0.02456247	0.2678647	2.04294157
s__Streptococcus anginosus	0	0.01462811	0.03552458	-0.4117744	0
s__Haemophilus influenzae	0.00694061	0.00607541	0.01894404	0.04567142	1.142410216
s__Lacticigenium naphtae	0.00110367	0.01641463	0.03159665	-0.4845755	0.067236821
s__Vagococcus salmoninarum	0.00244408	0.01552	0.03162222	-0.413504	0.157479618
s__Bifidobacterium thermacidophilum	0.00666652	0.00849122	0.01864817	-0.0978486	0.785107689
s__Halomonas campisalis	0.00681253	0.02109502	0.03664766	-0.3897243	0.322945199
s__Bacteroides coprophilus	0.00903757	0.00588715	0.015822	0.19911679	1.535136069
s__Coprococcus eutactus	0.01012463	0.00756297	0.01661389	0.15418809	1.338711565
s__Gracilibacillus halotolerans	0.00485723	0.01110639	0.01467777	-0.4257571	0.437336455
s__Alloiococcus otitis	0.00204273	0.01534042	0.02739296	-0.4854417	0.13315998
s__Rhodopseudomonas palustris	0	0.01150723	0.03696926	-0.3112649	0
s__Flavobacterium succinicans	0.00619719	0.03090401	0.09155367	-0.2698617	0.200530224
s__Shewanella algae	0.00732878	0.00528515	0.01158757	0.17636414	1.386674597
s__Porphyromonas endodontalis	0.0051209	0.00470244	0.01325743	0.03156406	1.08898751
s__Alistipes massiliensis	0.0010747	0.02231618	0.06193965	-0.3429382	0.048158006
s__Moellerella wisconsensis	0.00275917	0.00667975	0.02134572	-0.1836707	0.413064457
s__Thermomonas fusca	0.00041619	0.01616885	0.02200269	-0.7159424	0.025740429
s__Massilia haematophila	0.00138422	0.00495559	0.00717799	-0.4975443	0.279325122
s__Enterococcus asini	0.00426103	0.01267655	0.02396808	-0.3511136	0.336134855
s__Brevibacillus reuszeri	0.004009	0.00936755	0.02033743	-0.2634825	0.427966179
s__Kosmotoga mrcj	0.00607962	0.01022289	0.01412691	-0.2932892	0.594706514
s__Lactobacillus brevis	0.00890083	0.0080158	0.01356965	0.06522163	1.110411278
s__Micrococcus luteus	0.0081634	0.01142749	0.01750873	-0.1864262	0.714365313
s__Melissococcus plutonius	0.00393716	0.01801437	0.05043154	-0.2791351	0.218556752
s__Succinimonas amylolytica	0.00186422	0.01087156	0.01280954	-0.7031742	0.171476959
s__Erwinia chrysanthemi	0.00124858	0.01080193	0.02504216	-0.3814907	0.115588578
s__Photobacterium rosenbergii	0.00426103	0.01155207	0.01464899	-0.497716	0.368854341

s__Clostridium butyricum	0.00323142	0.00792774	0.01466064	-0.3203352	0.407609132
s__Corynebacterium durum	0.00317661	0.00658202	0.01448626	-0.2350786	0.482619322
s__Acinetobacter venetianus	0.00041619	0.01925713	0.03973981	-0.4741074	0.02161242
s__Thalassiosira pseudonana	0.00602742	0.00318011	0.00903437	0.31516416	1.895347741
s__Aeromonas sharmana	0.00154789	0.00838852	0.01225427	-0.558224	0.184524683
s__Parabacteroides gordonii	0.00238709	0.00801933	0.01143469	-0.4925576	0.297666874
s__Ochrobactrum intermedium	0	0.00863042	0.02772695	-0.3112649	0
s__Candidatus Phytoplasma witches-broom	0.01379432	0.00382951	0.01980699	0.5030959	3.602115174
s__Gluconacetobacter intermedius	0	0.00133223	0.00428005	-0.3112649	0
s__Clostridium sphenoides	0	0.0178009	0.05009856	-0.3553177	0
s__Lactobacillus delbrueckii	0.00317661	0.00800955	0.01953632	-0.2473823	0.396602725
s__Selenomonas ruminantium	0.00723089	0.01039087	0.02665275	-0.1185609	0.695889339
s__Burkholderia andropogonis	0.00110367	0.01300725	0.02386742	-0.4987377	0.084850198
s__Psychrobacter pulmonis	0.00254671	0.01789127	0.04089251	-0.3752414	0.142343641
Total	100	100	1.7256E-13	0	1

g__Bacteroides	34.9914724	22.025207	23.740636	0.54616335	1.588701183
g__Klebsiella	12.0934066	8.07321002	14.1369016	0.28437608	1.497967549
g__Proteus	2.2834271	3.91636058	9.23122804	-0.1768923	0.58304823
g__Enterococcus	2.68398767	5.29319468	10.5176109	-0.2480798	0.507063849
g__Dysgonomonas	1.39663458	2.90489844	6.82621032	-0.2209519	0.480786028
g__[Ruminococcus]	3.05313358	4.65800373	6.11185635	-0.2625831	0.655459668
g__Bifidobacterium	1.6041692	4.98391744	11.856474	-0.2850551	0.321869135
g__Oscillospira	3.67570445	2.58975827	4.08112312	0.26609003	1.419323375
g__Faecalibacterium	5.37520059	1.93518193	6.52372216	0.52730919	2.777620292
g__Coprococcus	1.0554944	2.19155304	2.61151262	-0.4350194	0.481619374
g__Blautia	1.74039944	2.98568999	3.35260297	-0.3714399	0.582913647
g__Pseudomonas	4.15093978	3.04863025	8.73033711	0.12626197	1.361575343
g__Escherichia	2.84677878	2.63815523	3.54998254	0.05876749	1.07907933

g__Acetobacter	0.01539907	0.44415556	1.28231645	-0.3343609	0.03467044
g__Parabacteroides	0.98546927	3.62390149	5.43287694	-0.4856418	0.271935999
g__Dorea	0.75280692	2.01746555	2.30303632	-0.5491267	0.373144872
g__Acinetobacter	0.57458755	1.97775374	2.9239899	-0.4798807	0.290525328
g__Ruminococcus	1.16735204	1.96302431	3.38644338	-0.2349581	0.594670188
g__Providencia	0.77708233	0.67813524	2.34164204	0.04225543	1.145910552
g__Roseburia	1.58238789	0.3549717	2.53429357	0.48432281	4.457786039
g__Megamonas	1.04735869	0.8130895	3.2342996	0.07243274	1.288122276
g__Enterobacter	0.76522595	0.5757523	0.85141378	0.22254004	1.329088835
g__Shigella	0.60489716	0.74177247	0.69259021	-0.1976281	0.815475345
g__Stenotrophomonas	0.20027796	0.94748913	2.06458399	-0.3619185	0.211377581
g__Citrobacter	0.56210956	0.76399698	0.83625554	-0.2414183	0.735748397
g__Serratia	0.49411145	0.9886822	1.68127926	-0.2941634	0.499767726
g__[Eubacterium]	0.67314613	0.80301819	1.30141128	-0.0997932	0.838270093
g__Erwinia	0.54412932	0.47567241	0.43792024	0.15632276	1.143916065
g__Dialister	0.42480753	0.6205731	1.71212251	-0.1143409	0.684540687
g__Lactococcus	0.01366152	0.66254547	1.80494778	-0.3595029	0.02061975
g__Lachnospira	0.8326838	0.24382432	1.22564025	0.48045051	3.415097436
g__Sutterella	0.04526552	1.74672621	3.71781878	-0.4576502	0.025914493
g__Akkermansia	0.79571249	0.65716094	1.8638781	0.07433509	1.210833514
g__Veillonella	0.68140489	0.53202909	1.38318074	0.10799442	1.280766222
g__Trabulsiella	0.19846632	0.34686114	0.46265953	-0.320743	0.572178014
g__Phascolarctobacterium	0.75735492	0.05748176	1.34367162	0.52086623	13.1755703
g__Prevotella	0.52939734	0.43789447	1.23730427	0.07395341	1.208961017
g__Ralstonia	0.36358911	0.71840095	1.40082241	-0.2532882	0.506108901
g__Streptococcus	0.0835369	0.36711642	0.39224131	-0.7229721	0.227548801
g__Comamonas	0.07878657	0.89012582	2.4453234	-0.3317922	0.08851172
g__Eggerthella	0.42195817	0.14292987	0.60560572	0.46074252	2.952204315
g__Bacillus	0.1505667	0.18597326	0.19765374	-0.1791343	0.809614759
g__Robinsoniella	0.00931155	0.25090977	0.69784345	-0.3462069	0.037111138

g__Solibacillus	0.13288724	0.16879063	0.21762037	-0.1649817	0.787290421
g__Lactobacillus	0.06728796	0.25130901	0.44631417	-0.4123128	0.267749894
g__Odoribacter	0.48169949	0.05110832	0.88386618	0.48716783	9.425070105
g__Collinsella	0.05865689	0.1767208	0.36648538	-0.3221517	0.331918454
g__Bilophila	0.28898864	0.20175208	0.53202558	0.16397062	1.432394869
g__Haemophilus	0.12499644	0.13634299	0.14868671	-0.0763118	0.916779362
g__Symbiobacterium	0.10982114	0.11431359	0.09794864	-0.0458654	0.960700587
g__Salmonella	0.13336872	0.06412831	0.14842944	0.46648699	2.079716528
g__Delftia	0.00202097	0.09980361	0.30082415	-0.3250492	0.020249457
g__Acidaminococcus	0.00421491	0.30508349	0.6645371	-0.4527491	0.013815587
g__Leuconostoc	0.00832625	0.34228056	0.74353435	-0.4491444	0.024325797
g__Moritella	0.09300212	0.08831924	0.09287521	0.05042122	1.053022207
g__Raoultella	0.05200816	0.07205749	0.12464907	-0.1608462	0.721759289
g__Nitrincola	0.07582716	0.0971728	0.14424166	-0.1479853	0.780333141
g__Holdemania	0.23913344	0.03839722	0.32048462	0.6263521	6.227884182
g__Staphylococcus	0.09357427	0.05692327	0.11839279	0.30957128	1.643866879
g__Porphyromonas	0.04200874	0.19197872	0.48984087	-0.3061606	0.21881976
g__Paenibacillus	0.05601641	0.06576547	0.04231978	-0.2303665	0.85176021
g__Pseudoramibacter_Eubacterium	0.17572038	0.02826407	0.22231794	0.66326772	6.217094869
g__Granulicatella	0.04443002	0.06001594	0.08209502	-0.1898522	0.740303624
g__Coprobacillus	0.10469993	0.06841326	0.15522856	0.23376286	1.53040409
g__Desulfovibrio	0.0140897	0.07013416	0.08324221	-0.6732697	0.200896445
g__Achromobacter	0.04238098	0.10000074	0.12435697	-0.4633416	0.423806688
g__Methanobrevibacter	0.21044795	0.0020022	0.43339029	0.48096544	105.1086042
g__Paraprevotella	0.16324212	0.00513967	0.33548165	0.47127004	31.76123592
g__Fusobacterium	0.00658996	0.01646843	0.03042111	-0.3247243	0.400156968
g__Shewanella	0.05076708	0.0418287	0.04919485	0.18169343	1.213690144
g__Corynebacterium	0.03790644	0.07484435	0.12534168	-0.2946977	0.506470282
g__Morganella	0.02783066	0.03404284	0.05424262	-0.1145258	0.817518725
g__Plesiomonas	0.02104928	0.04249844	0.08182326	-0.2621402	0.495295255

g__Epulopiscium	0.09677114	0.01859085	0.16642051	0.46977558	5.205311208
g__Anaerotruncus	0.08443687	0.01990447	0.1273583	0.50669964	4.242106976
g__Finegoldia	0.00062125	0.08745997	0.26396651	-0.3289763	0.007103226
g__Pseudoxanthomonas	0.02877718	0.04173436	0.03555705	-0.3644054	0.689532105
g__Adlercreutzia	0.07583568	0.01855839	0.12396532	0.46204286	4.086328067
g__[Prevotella]	0.0150695	0.01894921	0.02328194	-0.1666405	0.795257191
g__Anaerostipes	0.02893353	0.02684361	0.03640532	0.05740701	1.077855427
g__Aquamonas	0.02765701	0.03262693	0.04951679	-0.1003683	0.847674412
g__Cronobacter	0.02563986	0.03014304	0.04133714	-0.1089378	0.850606379
g__Actinomyces	0.0033354	0.11025656	0.3143844	-0.3400969	0.030251299
g__Sarcina	0.01491115	0.03577903	0.06748892	-0.3092046	0.416756639
g__Janthinobacterium	0.01367262	0.02290333	0.03199	-0.2885499	0.59697075
g__Pseudoalteromonas	0.02567825	0.01961259	0.02076352	0.29213084	1.309274136
g__Psychrobacter	0.00968204	0.08790135	0.24088436	-0.3247173	0.110146636
g__Flavobacterium	0.01283106	0.07617901	0.19775429	-0.3203367	0.168432981
g__Campylobacter	0.03150533	0.02333759	0.08109967	0.10071232	1.349981916
g__Acetobacterium	0.01245432	0.0233384	0.0174411	-0.6240484	0.533640403
g__Devosia	0.02631773	0.01935811	0.02545487	0.27340998	1.359519343
g__Halomonas	0.02116522	0.02964301	0.03252481	-0.260656	0.714003783
g__Tatumella	0.01506839	0.01535612	0.02355262	-0.0122163	0.981263139
g__Desulfosporosinus	0.01732208	0.01849228	0.0141297	-0.0828184	0.936719622
g__Vibrio	0.02241197	0.01682926	0.02318514	0.2407881	1.331726101
g__Megasphaera	0.00528385	0.04567339	0.10246389	-0.3941831	0.115687804
g__Anaerococcus	0.00589954	0.02534173	0.03746917	-0.518885	0.232799423
g__Nesterenkonia	0.01649148	0.03020906	0.04353043	-0.3151264	0.545911519
g__Acinetobacter	0.0125402	0.00933546	0.00674806	0.47491156	1.343285917
g__Vagococcus	0.01445686	0.01281683	0.03570789	0.04592925	1.127959644
g__Aeromonas	0.0157353	0.01551186	0.01581619	0.01412724	1.014404405
g__Neisseria	0.01364265	0.01130007	0.02027837	0.11552155	1.207307523
g__Thauera	0.00438833	0.02242018	0.03036254	-0.5938846	0.195731439

g__Peptoniphilus	0.01276615	0.03611936	0.07016222	-0.332846	0.353443302
g__WAL_1855D	0.00889221	0.01782341	0.01246776	-0.7163435	0.498906336
g__Anoxybacillus	0.01965577	0.01195865	0.01602723	0.48025292	1.643644941
g__Candidatus Phytoplasma	0.01261791	0.01557944	0.01191416	-0.248572	0.809907933
g__Candidatus Portiera	0.01514539	0.01596417	0.01509761	-0.0542321	0.948711649
g__Paucibacter	0	0.01465386	0.04484067	-0.3267985	0
g__Ochrobactrum	0.00596695	0.02202581	0.02631466	-0.610263	0.270907153
g__Dictyostelium	0.00899228	0.01059359	0.00642111	-0.2493816	0.84884197
g__Butyrivibrio	0.01262462	0.0123756	0.01182792	0.02105292	1.020121221
g__Rhodobacter	0	0.01422426	0.04569825	-0.3112649	0
g__Turicibacter	0.00374238	0.0251935	0.05172833	-0.414688	0.14854553
g__Pseudobutyrivibrio	0.01028857	0.01229078	0.01818897	-0.1100784	0.837096366
g__Paludibacter	0.01335295	0.00824081	0.01198187	0.42665606	1.620343535
g__Burkholderia	0.00972842	0.01743886	0.01655765	-0.4656719	0.557859015
g__Lysinibacillus	0.01231627	0.01538453	0.02705147	-0.1134232	0.800561694
g__Swaminathania	0.00234999	0.0037099	0.00736322	-0.1846889	0.633438753
g__Streptomyces	0.01118674	0.01514782	0.02023719	-0.1957327	0.738505023
g__Glaciecola	0.01061678	0.01140265	0.01150743	-0.0682926	0.931079859
g__Pantoea	0.01090106	0.0089705	0.01296245	0.14893552	1.215213198
g__Geodermatophilus	0.0060911	0.01388207	0.02865746	-0.2718652	0.438774736
g__Natronobacillus	0.00841232	0.01013857	0.01423953	-0.1212292	0.829734604
g__Lactonifactor	0.03862123	0.00358729	0.05441622	0.64381417	10.76612201
g__Lachnobacterium	0.00498179	0.01583278	0.02708972	-0.4005575	0.314650158
g__Marinobacter	0.01058596	0.01056741	0.01515024	0.00122478	1.001755936
g__Histophilus	0.00873332	0.01101492	0.01353416	-0.1685807	0.792862925
g__Brenneria	0.00985581	0.00836198	0.01277011	0.11697894	1.178646046
g__Enhydrobacter	0.01208307	0.00816653	0.01291385	0.3032818	1.479583446
g__Trichococcus	0.00539164	0.00946302	0.01792946	-0.2270776	0.569759131
g__Rheinheimera	0.01018887	0.01039734	0.01223323	-0.0170412	0.979949804
g__Abiotrophia	0.001713	0.00844784	0.0247239	-0.2724022	0.202773365

g__Sporosarcina	0.00187316	0.01549484	0.02752464	-0.4948907	0.120888934
g__Marinobacterium	0.00885788	0.00495816	0.00716403	0.54434717	1.786525527
g__Kaistobacter	0.01075787	0.00850148	0.00941596	0.23963462	1.265411493
g__Desulfotomaculum	0.00859127	0.0109739	0.01403745	-0.1697338	0.782881977
g__Rhodobacter	0.00991503	0.00824091	0.00744969	0.224724	1.20314796
g__Moryella	0.01050865	0.00890798	0.01080779	0.14810364	1.179689873
g__Anaerobaculum	0.01070306	0.01013232	0.01062322	0.05372557	1.056328541
g__Christensenella	0.01768514	0.01279108	0.02916483	0.16780686	1.382614936
g__Jeotgalicoccus	0.00034267	0.02802136	0.06227536	-0.4444567	0.012228795
g__Limnohabitans	0.00234435	0.01111349	0.02019893	-0.4341389	0.210946309
g__Euzebya	0.01022819	0.00604418	0.00686877	0.60913357	1.692235813
g__Gemella	0.00382578	0.00943063	0.0128097	-0.4375471	0.405675848
g__Methylophaga	0.00877284	0.00704458	0.00934606	0.18491919	1.245332697
g__Dickeya	0.01076869	0.00856646	0.01458054	0.15103919	1.257076205
g__Methylibium	0.00181535	0.00797511	0.01783857	-0.3453062	0.227626309
g__Pyramidobacter	0	0.05041297	0.16196164	-0.3112649	0
g__Allobaculum	0.0081255	0.00835411	0.00602601	-0.0379384	0.97263416
g__Candidatus Azobacteroides	0.00669609	0.00428817	0.00703491	0.34228157	1.561525347
g__Desulfococcus	0.00769142	0.00608663	0.00642512	0.24976857	1.263658418
g__HTCC	0.00597739	0.00684758	0.00911771	-0.0954388	0.872920953
g__Sphingobacterium	0.00382321	0.00815703	0.01188946	-0.3645093	0.468701495
g__Aggregatibacter	0.0101661	0.00228162	0.00755206	1.04401694	4.765645339
g__Alcaligenes	0.00121982	0.00804582	0.02259229	-0.3021385	0.151608763
g__Bulleidia	0.00156172	0.0055994	0.00972966	-0.4149858	0.278909512
g__Halorhodospira	0.00761472	0.00525391	0.01147596	0.20571782	1.44934309
g__Novosphingobium	0.0067295	0.00716606	0.00695533	-0.0627667	0.93907906
g__Brevundimonas	0.00156148	0.02553365	0.06764744	-0.3543692	0.061153658
g__Bradyrhizobium	0.00658462	0.00589221	0.008051	0.08600305	1.117512937
g__Butyricimonas	0.01477663	0.00242915	0.02587738	0.47715318	6.083043813
g__Rhodoferax	0.0027662	0.00593964	0.01047054	-0.3030831	0.465718216

g__Candidatus Phlomobacter	0.00495583	0.00374264	0.00688422	0.17622813	1.324154524
g__Zoogloea	0.00582918	0.00712303	0.01019149	-0.1269536	0.818357276
g__Arthrobacter	0.00373125	0.00846305	0.01093771	-0.4326133	0.440887207
g__Alistipes	0.00162713	0.0093097	0.01540444	-0.4987245	0.174777868
g__Sphingomonas	0.00457216	0.00805415	0.01019933	-0.3413939	0.567677566
g__Listeria	0.002838	0.00457898	0.00894408	-0.194652	0.619787844
g__Oleispira	0.00509607	0.00679768	0.01414254	-0.1203183	0.749678355
g__Clostridium	0.00093776	0.00981422	0.01009446	-0.8793403	0.095551018
g__Marinimicrobium	0.00643543	0.00468526	0.00897413	0.19502363	1.373547431
g__Gluconacetobacter	0.00528696	0.00523142	0.00680202	0.0081652	1.010616601
g__Alkalimonas	0.00290232	0.00661041	0.00772483	-0.4800224	0.439052531
g__Selenomonas	0.00811821	0.00220434	0.01130395	0.5231693	3.682839926
g__Oribacterium	0.00194755	0.00384036	0.00569103	-0.3325962	0.507126227
g__Leucothrix	0.00666076	0.00606409	0.0091152	0.06545877	1.098393863
g__Weissella	0.00352699	0.00484521	0.00485832	-0.2713324	0.727933335
g__Agrobacterium	0.00340842	0.0051966	0.00638662	-0.2799896	0.655892944
g__Mogibacterium	0.00091135	0.02850759	0.08616006	-0.3202904	0.031968794
g__Filifactor	0.00424255	0.00598365	0.00823237	-0.2114941	0.70902427
g__Mycoplana	0.00372557	0.00700288	0.00769304	-0.4260091	0.532006053
g__Alloiococcus	0.00440391	0.00456203	0.00706309	-0.0223868	0.965340005
g__Sodalis	0.00593555	0.00397609	0.00618624	0.31674433	1.492809286
g__Bdellovibrio	0.00305684	0.0035363	0.00356091	-0.1346436	0.864419383
g__Roseateles	0	0.00522435	0.01381041	-0.3782903	0
g__Candidatus Hamiltonella	0.00476923	0.00333291	0.00573805	0.25031494	1.430951291
g__Cedecea	0.0080893	0.00287369	0.01075269	0.48505185	2.814954958
g__Candidatus Regiella	0.00481375	0.00513837	0.0079098	-0.0410403	0.936824254
g__Succinivibrio	0.00222785	0.00475159	0.00563869	-0.4475757	0.468863892
g__Ramlibacter	0.00133718	0.00657532	0.00599423	-0.8738644	0.203362986
g__Slackia	0.00377283	0.00469061	0.00562934	-0.1630351	0.8043367
g__Photorhabdus	0.00376861	0.0028255	0.00427604	0.22055755	1.33378662

g__Leclercia	0.00254077	0.00276666	0.00515538	-0.0438162	0.918353201
g__Moraxella	0.00433321	0.00573825	0.0060094	-0.2338069	0.75514518
g__Pseudidiomarina	0.00272741	0.0039606	0.00532361	-0.2316456	0.688635267
g__Photobacterium	0.00410135	0.00365384	0.00565457	0.0791412	1.122476615
g__Limnobacter	0.00237456	0.00684047	0.0113457	-0.3936216	0.347134087
g__Facklamia	0.00457059	0.0049189	0.00950931	-0.0366281	0.929189835
g__Fusibacter	0.00153713	0.00370719	0.00552392	-0.3928485	0.414634259
g__Planococcus	0.00329987	0.0031815	0.00403923	0.02930738	1.037208654
g__Lysobacter	0.00365386	0.00356752	0.00513342	0.01682011	1.024203005
g__Schneideria	0.00410695	0.00306186	0.00455799	0.22928693	1.341324157
g__Alicyclobacillus	0.00195033	0.00310374	0.00374719	-0.3078068	0.628380394
g__Alteromonas	0.00310505	0.00347156	0.00474054	-0.077316	0.894422406
g__Thiothrix	0.00289387	0.00310462	0.00357375	-0.0589727	0.932116214
g__Gluconacetobacter	5.9559E-05	0.00083731	0.00207592	-0.3746549	0.071130819
g__Tolumonas	0.0049372	0.00174353	0.00549801	0.58087707	2.831727552
g__Nitrospira	0.00246092	0.00280109	0.00415828	-0.0818036	0.878560447
g__Rothia	0.0035956	0.0037678	0.00860057	-0.0200217	0.954297325
g__Treponema	0.00267027	0.00350156	0.00373209	-0.2227398	0.762595386
g__Gracilibacter	0.0042428	0.00243541	0.00465871	0.38795893	1.742129006
g__Polaribacter	0.00208061	0.00241638	0.0031725	-0.1058404	0.861040957
g__Mobiluncus	0	0.01883343	0.05667379	-0.3323129	0
g__Borrelia	0.00094117	0.00284622	0.00393165	-0.4845442	0.330671729
g__Legionella	0.00159147	0.00276193	0.00574878	-0.2036013	0.576216959
g__Marinococcus	0.00120899	0.00147301	0.00205473	-0.128494	0.820761522
g__Variovorax	0	0.00417345	0.00697877	-0.5980203	0
g__Profftia	0.00210889	0.00235961	0.00477418	-0.0525161	0.893744495
g__Brevibacillus	0.0018758	0.00174527	0.00328614	0.03972183	1.074791761
g__Dechloromonas	0.00101873	0.00260431	0.00597069	-0.2655612	0.391169301
g__Ferrimonas	0.00337897	0.00356922	0.00534892	-0.0355672	0.946698066
g__Haloferula	0.00636856	0.00273321	0.01439352	0.25256891	2.33006961

g__Acidaminobacter	0.00223833	0.00206455	0.00329968	0.05266555	1.08417294
g__Thalassomonas	0.00984468	0.00168986	0.01898093	0.42963227	5.825742339
g__Leptothrix	0.00028311	0.00403641	0.00592678	-0.6332779	0.070138764
g__Bombiscardovia	0.00194861	0.00324807	0.00738006	-0.1760772	0.599928765
g__Edwardsiella	0.00235193	0.00385131	0.00695362	-0.2156262	0.610682433
g__Cellvibrio	0.00179644	0.00421628	0.00684985	-0.353269	0.42607228
g__Asticcacaulis	0.00046178	0.00058708	0.0014248	-0.0879381	0.78657993
g__Azorhizophilus	0.0021311	0.00360491	0.00609611	-0.2417637	0.591164074
g__Geobacillus	0.00357255	0.00296724	0.00547255	0.11060793	1.203996497
g__Rhodoplanes	0.0013665	0.00236798	0.0033505	-0.2989042	0.577073448
g__Rummeliibacillus	0.00160092	0.00305983	0.00384349	-0.3795795	0.523204832
g__Rahnella	0.00138652	0.00261548	0.00283119	-0.4340782	0.530121628
g__Tannerella	0.00363206	0.00190412	0.00491589	0.35149968	1.907470117
g__Dehalobacterium	0.0016101	0.00298082	0.0082756	-0.165634	0.540153527
g__Succiniclasticum	0.00235271	0.00243865	0.00407937	-0.021066	0.964760771
g__Arcobacter	0.00189027	0.00351861	0.00419452	-0.3882063	0.537220858
g__Octadecabacter	0.00271518	0.00113527	0.0027036	0.58437047	2.391649601
g__Acidithiobacillus	0.00197574	0.00285967	0.00329796	-0.268023	0.690898165
g__Anaerobacillus	0.00151729	0.00279729	0.00484085	-0.2644159	0.542414627
g__Desulfovermiculus	0.00310983	0.002812	0.00395599	0.0752859	1.105914236
g__Micrococcus	0.0019798	0.00321426	0.00369293	-0.334276	0.615943565
g__Pectinatus	0.00389426	0.00126407	0.00892147	0.29481565	3.080730466
g__Dokdonella	0.0004578	0.00289666	0.00367406	-0.6638069	0.158042553
g__Proteiniclasticum	0.00183136	0.00182778	0.00355168	0.00100751	1.001957747
g__human	0.00731376	0.00182075	0.01439873	0.38149235	4.016889768
g__Anaerovibrio	0.00346284	0.00168313	0.00414998	0.42884779	2.057380077
g__Polaromonas	0.00168422	0.0027061	0.00380912	-0.2682721	0.622378732
g__Buchnera	0.00130459	0.00310823	0.00510309	-0.3534394	0.419722537
g__Gordonia	0.00186233	0.00201098	0.00342715	-0.0433747	0.926080208
g__Shuttleworthia	0.00121777	0.00131262	0.00282196	-0.0336095	0.927743686

g__Polynucleobacter	0.00158794	0.00179475	0.00294238	-0.070286	0.884770344
g__Fimbriimonas	0.00227531	0.00204568	0.00350702	0.06547742	1.112251425
g__Pediococcus	0.00101063	0.00266668	0.00449968	-0.3680366	0.378984457
g__Xanthomonas	0.00048413	0.00365468	0.00496679	-0.638351	0.132468282
g__Kordia	0.00211131	0.00185077	0.00304733	0.08549777	1.14077391
g__Reinekea	0.00037786	0.003046	0.00922772	-0.2891437	0.124050306
g__Thiovirga	0.00198502	0.0019968	0.00407471	-0.0028906	0.994101329
g__Lacticigenium	0.00048678	0.00224546	0.00283507	-0.620329	0.216785651
g__Candidatus Solibacter	0.00178715	0.00121393	0.00306617	0.18695001	1.472201812
g__Ruminobacter	0.00349194	0.00130401	0.00377944	0.57890302	2.677844876
g__Anaerofustis	0.00307446	0.00207174	0.00428247	0.23414426	1.483995985
g__Gracilibacillus	0.00068133	0.00250173	0.00269023	-0.6766719	0.272344958
g__Rhodopseudomonas	0	0.00220959	0.00709876	-0.3112649	0
g__Thermicanus	0.00063571	0.00285572	0.00447608	-0.4959717	0.222610212
g__Thermomonas	0.00005760	0.00330611	0.00169428	-0.8788073	0.018014689
g__Candidatus Blochmannia	0.00246841	0.00205003	0.00428517	0.09763577	1.204088104
g__Moellerella	0.00121696	0.00140115	0.00464855	-0.0396243	0.868540086
g__Catenibacterium	0.00250261	0.00202434	0.00362199	0.13204744	1.23626198
g__Exiguobacterium	0.00397195	0.00167814	0.00339937	0.96894665	6.247088755
g__Propionigenium	0.0012634	0.00158512	0.00208837	-0.1540512	0.797040142
g__Rubrobacter	0.00262596	0.00346586	0.00637586	-0.1317316	0.757664182
g__Pelomonas	0.00021955	0.00401514	0.00854668	-0.4441012	0.054680514
g__Kosmotoga	0.00145921	0.00136129	0.00225751	0.04337611	1.071933166
g__Leptospira	0.00055822	0.00279052	0.00394668	-0.5656138	0.200040873
g__Massilia	0.00036251	0.00199525	0.00372291	-0.4385672	0.181685495
g__Chryseobacterium	0.00138699	0.00164866	0.00298882	-0.0875491	0.841283897
g__Desulfitobacter	0	0.00967043	0.02965394	-0.3261093	0
g__Actinobacillus	0.00091559	0.00157486	0.00336743	-0.1957791	0.581378386
g__Melissococcus	0.00084933	0.0015008	0.00386505	-0.1685552	0.565915545
g__Oceanobacillus	0.00100541	0.00263193	0.00444539	-0.3658882	0.382006201

g__Parvimonas	0.00045704	0.00414789	0.00835515	-0.4417458	0.110185245
g__Gallicola	0.00112576	0.00520229	0.01343167	-0.3035018	0.216396404
g__Perlucidibaca	0.0008969	0.00158035	0.00210236	-0.3250874	0.567531093
g__Candidatus Cardinium	0.00229839	0.00191081	0.00466577	0.08306928	1.202836509
g__Carnobacterium	0.00080961	0.0014692	0.00328703	-0.2006635	0.551055919
g__Mycobacterium	0.00108032	0.00229041	0.0034866	-0.3470674	0.471671459
g__Caloramator	0.00288831	0.00113108	0.00405511	0.43333864	2.553598398
g__Geobacter	0	0.00346104	0.00259735	-1.0245191	0
g__Sporanaerobacter	0.00155626	0.00191561	0.00349409	-0.1028466	0.812407245
g__Marinomonas	0.0006366	0.00208747	0.00334008	-0.4343819	0.304961062
g__Salinicoccus	0.00084933	0.00137524	0.00305362	-0.1722278	0.617582298
g__Succinimonas	0.0005265	0.00259329	0.00325773	-0.6344262	0.203024268
g__Alcanivorax	0.00310251	0.00071056	0.00360039	0.66435707	4.366273021
g__Aequorivita	0.00134808	0.00140939	0.00273787	-0.0223943	0.95649711
g__Desemzia	0.00098679	0.00093706	0.00273773	0.01816487	1.053070871
g__Schwartzia	0.00194713	0.00126407	0.0056266	0.12139834	1.540365233
g__Thalassiosira	0.00108579	0.00075999	0.00195906	0.16630441	1.428689577
g__Yersinia	0.00051735	0.0009312	0.00160485	-0.257871	0.5555783
g__Halochromatium	0.0011947	0.00139824	0.0026059	-0.0781066	0.854432701
g__Thermoanaerobacterium	0.00162251	0.00188314	0.00326401	-0.0798492	0.861599086
g__Candidatus Accumulibacter	0.00121854	0.00237646	0.00382288	-0.3028903	0.512755942
g__Chelonobacter	0.00162116	0.00177647	0.00412205	-0.0376762	0.91257749
g__Deinococcus	0.00240105	0.00022175	0.00212391	1.02607997	11.52787729
g__Herminiimonas	0.00024339	0.00348187	0.00420384	-0.7703608	0.069902525
g__Salinispora	0.00182815	0.00184808	0.00532384	-0.0037443	0.989213724
g__Methylobacterium	0.00078124	0.00102989	0.00162842	-0.1526955	0.758564716
g__Microbacterium	0.00064412	0.00291785	0.00499013	-0.4556453	0.22075186
g__Aerococcus	0.00108357	0.00390575	0.01078268	-0.2617324	0.277430066
g__Oceanimonas	0.00162327	0.00135577	0.00291246	0.09184727	1.19730614
g__Peptostreptococcus	0.00067512	0.0019667	0.00330801	-0.3904397	0.343276035

Total	100	100	2.4848E-13	-0.1715713	1
p__Proteobacteria	40.3547498	42.4801331	23.9301636	-0.0888161	0.949967594
p__Firmicutes	30.5608052	33.3401721	20.1618959	-0.1378525	0.916636098
p__Bacteroidetes	27.2485774	20.6696977	18.2284264	0.3609132	1.318286209
p__Actinobacteria	1.20767149	2.82081323	5.68597883	-0.2837052	0.42812884
p__Verrucomicrobia	0.31669619	0.49142405	1.23079384	-0.1419635	0.644445851
p__Euryarchaeota	0.13167979	0.00109544	0.27155262	0.48088046	120.2076932
p__Fusobacteria	0.00503968	0.01275379	0.02339937	-0.3296717	0.395151432
p__Chloroflexi	0.02626083	0.03034841	0.01985888	-0.2058312	0.865311669
p__Acidobacteria	0.02847101	0.02387803	0.01357189	0.33841868	1.19235182
p__Tenericutes	0.05242055	0.01978264	0.07773238	0.41987544	2.649826544
p__Planctomycetes	0.0131611	0.01891482	0.00881364	-0.6528209	0.695808703
p__Spirochaetes	0.00712595	0.01510751	0.00665385	-0.8989606	0.543654435
p__Synergistetes	0.0100821	0.03552211	0.09289061	-0.2738707	0.283826007
p__Cyanobacteria	0.00858942	0.00991109	0.00625941	-0.2111483	0.866648008
p__Crenarchaeota	0.00945869	0.0095788	0.00858831	-0.0139858	0.987460411
p__Gemmatimonadetes	0.00560375	0.00802877	0.0063993	-0.3789507	0.697958838
p__Armatimonadetes	0.00482294	0.0038064	0.00457811	0.22204335	1.267060663
p__Fibrobacteres	0.00415098	0.00586691	0.00720625	-0.2381174	0.707523429
p__Nitrospirae	0.00310399	0.00314886	0.00317845	-0.0141191	0.985748214
p__Elusimicrobia	0.0007977	0.0012949	0.0018972	-0.2620709	0.616030289
p__Thermotogae	0.00073138	0.00072139	0.00108903	0.0091754	1.01385145

Supplemental Table S2 – P value difference in the mean relative abundance (RA) between blenderized tube feeds (blended group) and on standard enteral formula (control) groups at species, genus, and phylum levels.

Elevated in	Taxa	Blended Vs. Control (p-Value)
	**At Species level**	
Blended	s__Bacteroides caccae	**0.0222**
Blended	s__Pseudoalteromonas piscicida	**0.0468**
Blended	s__Enterobacter nickellidurans	**0.0486**
Blended	s__Bacteroides uniformis	0.0687
Control	s__Thermomonas fusca	0.0727
Blended	s__Klebsiella oxytoca	0.0795
Blended	s__Lactonifactor longoviformis	0.0819
Control	s__Succinimonas amylolytica	0.0824
Control	s__Parabacteroides distasonis	0.0953
Control	s__Dorea formicigenerans	0.1064
Blended	s__Salmonella enterica	0.1093
Control	s__Blautia producta	0.1115
Blended	s__Clostridium lavalense	0.1135
Blended	s__Eggerthella lenta	0.1243
Blended	s__Enterobacter ludwigii	0.1301
Blended	s__Aeromonas caviae	0.1378
Blended	s__Enterobacter cloacae	0.1417
Blended	s__[Clostridium] irregulare	0.1431
Blended	s__Cedecea neteri	0.1461
Blended	s__Shewanella benthica	0.1592
Blended	s__Erwinia soli	0.1605

Control	s__[Ruminococcus] gnavus	0.1611
Control	s__Aeromonas sharmana	0.1722
Control	s__Bacillus halodurans	0.1734
Blended	s__Candidatus Phytoplasma witches-broom	0.1772
Blended	s__Bacillus muralis	0.1823
Control	s__Clostridium spiroforme	0.1833
Blended	s__Campylobacter ureolyticus	0.1838
Blended	s__Neisseria subflava	0.1979
Blended	s__Enterobacter hormaechei	0.2004
Blended	s__Aeromonas hydrophila	0.2021
Blended	s__Enterobacter cancerogenus	0.2078
Control	s__Enterococcus casseliflavus	0.2091
Blended	s__Brenneria nigrifluens	0.2142
Blended	s__Bacillus thermoamylovorans	0.2152
Blended	s__Clostridium methylpentosum	0.2182
Control	s__Burkholderia andropogonis	0.2197
Blended	s__Schneideria nysicola	0.2215
Blended	s__Bacteroides acidifaciens	0.2223
Blended	s__Bacillus horneckiae	0.2252
Control	s__Massilia haematophila	0.2302
Control	s__Stenotrophomonas rhizophila	0.2326
Control	s__Lacticigenium naphtae	0.2327
Control	s__Alloiococcus otitis	0.2344
Control	s__Parabacteroides gordonii	0.2362
Control	s__Photobacterium rosenbergii	0.2367
Control	s__Acinetobacter venetianus	0.2416
Blended	s__Serratia ureilytica	0.2558
Control	s__Bifidobacterium animalis	0.2620
Blended	s__Acinetobacter radioresistens	0.2623
Blended	s__Enterobacter aerogenes	0.2638

Blended	s__Trabulsiella farmeri	0.2643
Control	s__Desulfovibrio oxamicus	0.2681
Control	s__Bacillus endophyticus	0.2782
Control	s__Bacillus cereus	0.2921
Control	s__Acinetobacter guillouiae	0.2931
Control	s__Clostridium hathewayi	0.2939
Control	s__Acinetobacter johnsonii	0.2974
Blended	s__Klebsiella pneumoniae	0.2979
Control	s__Weissella viridescens	0.2993
Control	s__Collinsella stercoris	0.3005
Control	s__Clostridium saccharolyticum	0.3016
Blended	s__Tatumella punctata	0.3059
Control	s__Streptococcus anginosus	0.3099
Control	s__Vagococcus salmoninarum	0.3136
Control	s__Gracilibacillus halotolerans	0.3190
Blended	s__Clostridium perfringens	0.3214
Control	s__Acinetobacter schindleri	0.3233
Control	s__Bulleidia moorei	0.3276
Blended	s__Enterobacter amnigenus	0.3284
Control	s__Variovorax paradoxus	0.3445
Control	s__Erwinia chrysanthemi	0.3514
Control	s__Halomonas campisalis	0.3520
Control	s__Psychrobacter pulmonis	0.3605
Control	s__Streptococcus luteciae	0.3608
Control	s__Alkalimonas amylolytica	0.3658
Control	s__Brevundimonas diminuta	0.3680
Control	s__Clostridium sphenoides	0.3819
Blended	s__Thalassiosira pseudonana	0.3847
Control	s__Clostridium citroniae	0.3851
Control	s__Staphylococcus equorum	0.3861

Blended	s__Enterobacter radicincitans	0.3867
Control	s__Robinsoniella peoriensis	0.3872
Blended	s__Clostridium difficile	0.3892
Blended	s__Faecalibacterium prausnitzii	0.3923
Blended	s__human gut metagenome	0.3954
Control	s__[Eubacterium] biforme	0.3964
Control	s__Alistipes massiliensis	0.4004
Control	s__Enterococcus asini	0.4031
Control	s__Roseateles depolymerans	0.4058
Control	s__Stenotrophomonas acidaminiphila	0.4191
Control	s__Lactobacillus plantarum	0.4208
Control	s__Lactobacillus zeae	0.4214
Control	s__Clostridium hiranonis	0.4222
Control	s__[Clostridium] sordellii	0.4224
Blended	s__Rothia mucilaginosa	0.4236
Control	s__Brenneria quercina	0.4251
Control	s__Lysinibacillus boronitolerans	0.4259
Control	s__Enterobacter cowanii	0.4331
Blended	s__Shewanella hanedai	0.4334
Control	s__Bifidobacterium bifidum	0.4371
Blended	s__Shigella flexneri	0.4377
Control	s__Alcaligenes faecalis	0.4406
Control	s__Clostridium aerotolerans	0.4422
Control	s__Listeria weihenstephanensis	0.4431
Control	s__Rhodobacter sphaeroides	0.4444
Control	s__Pyramidobacter piscolens	0.4444
Control	s__Rhodopseudomonas palustris	0.4444
Control	s__Ochrobactrum intermedium	0.4444
Control	s__Gluconacetobacter intermedius	0.4444
Control	s__Clostridium bartlettii	0.4458

Control	s__Alistipes indistinctus	0.4480
Control	s__Acinetobacter lwoffii	0.4508
Control	s__Clostridium butyricum	0.4513
Blended	s__Bifidobacterium breve	0.4529
Blended	s__Haloferula harenae	0.4549
Control	s__Dictyostelium citrinum	0.4557
Control	s__Citrobacter gillenii	0.4566
Control	s__Clostridium clostridioforme	0.4589
Control	s__Rahnella aquatilis	0.4616
Control	s__Serratia marcescens	0.4635
Blended	s__Brenneria alni	0.4639
Control	s__Serratia rubidaea	0.4753
Blended	s__Bacteroides ovatus	0.4810
Control	s__Clostridium aldenense	0.4814
Control	s__Clostridium neonatale	0.4833
Blended	s__Pseudomonas umsongensis	0.4863
Blended	s__Shigella boydii	0.4886
Control	s__Melissococcus plutonius	0.5007
Blended	s__Akkermansia muciniphila	0.5016
Blended	s__Leclercia adecarboxylata	0.5053
Control	s__Pseudomonas fragi	0.5091
Blended	s__Aeromonas sobria	0.5093
Control	s__Kosmotoga mrcj	0.5134
Control	s__Flavobacterium succinicans	0.5143
Blended	s__Candidatus Regiella insecticola	0.5191
Control	s__Desulfosporosinus meridiei	0.5252
Control	s__Roseburia faecis	0.5331
Control	s__Cronobacter dublinensis	0.5357
Control	s__Collinsella aerofaciens	0.5361
Control	s__Planococcus maitriensis	0.5369

Control	s__Brevibacillus reuszeri	0.5374
Blended	s__Pseudidiomarina homiensis	0.5402
Blended	s__Bacteroides eggerthii	0.5433
Blended	s__Veillonella dispar	0.5501
Control	s__Blautia obeum	0.5526
Control	s__Stenotrophomonas geniculata	0.5599
Control	s__Lactobacillus delbrueckii	0.5603
Control	s__Pseudoxanthomonas indica	0.5603
Blended	s__Shigella sonnei	0.5613
Control	s__Haemophilus parainfluenzae	0.5626
Blended	s__Bacteroides coprophilus	0.5696
Control	s__Bacillus flexus	0.5707
Control	s__Prevotella copri	0.5813
Blended	s__Dysgonomonas gadei	0.5865
Control	s__Corynebacterium durum	0.5868
Blended	s__Ruminococcus flavefaciens	0.5940
Blended	s__Citrobacter freundii	0.5945
Control	s__Bifidobacterium adolescentis	0.5991
Blended	s__Shewanella algae	0.6024
Blended	s__Escherichia coli	0.6029
Blended	s__Cronobacter sakazakii	0.6319
Control	s__[Ruminococcus] torques	0.6330
Blended	s__Coprococcus eutactus	0.6431
Control	s__Pseudomonas stutzeri	0.6442
Control	s__Pseudomonas viridiflava	0.6499
Blended	s__Pantoea agglomerans	0.6547
Control	s__Pseudomonas nitroreducens	0.6630
Control	s__Asticcacaulis biprosthecium	0.6662
Control	s__Moellerella wisconsensis	0.6666
Control	s__Bradyrhizobium elkanii	0.6873

Blended	s__Clostridium tertium	0.6934
Control	s__Micrococcus luteus	0.6988
Control	s__Prevotella stercorea	0.7025
Blended	s__[Clostridium] difficile	0.7026
Blended	s__Veillonella parvula	0.7079
Control	s__Bacteroides plebeius	0.7098
Control	s__Histophilus somni	0.7109
Blended	s__Erwinia dispersa	0.7190
Control	s__Coprobacillus cateniformis	0.7292
Control	s__Clostridium celatum	0.7355
Blended	s__Bifidobacterium longum	0.7368
Blended	s__Plesiomonas shigelloides	0.7437
Blended	s__Pseudomonas veronii	0.7479
Control	s__Enterococcus haemoperoxidus	0.7492
Control	s__Staphylococcus aureus	0.7549
Blended	s__Staphylococcus epidermidis	0.7590
Control	s__Bacteroides fragilis	0.7625
Blended	s__Acinetobacter rhizosphaerae	0.7743
Blended	s__Lactobacillus brevis	0.8008
Control	s__Pseudomonas citronellolis	0.8019
Control	s__Selenomonas ruminantium	0.8022
Blended	s__Janthinobacterium lividum	0.8107
Blended	s__Escherichia hermannii	0.8497
Control	s__Bifidobacterium thermacidophilum	0.8514
Control	s__Pseudomonas mendocina	0.8548
Control	s__Raoultella ornithinolytica	0.8583
Control	s__Morganella morganii	0.8588
Control	s__[Eubacterium] dolichum	0.8647
Blended	s__Haemophilus influenzae	0.8697
Blended	s__Prevotella nigrescens	0.8838

Control	s__Citrobacter youngae	0.8880
Blended	s__Anoxybacillus kestanbolensis	0.8894
Blended	s__Porphyromonas endodontalis	0.8946
Control	s__Halorhodospira halophila	0.9016
Control	s__Aquamonas haywardensis	0.9022
Control	s__Pseudomonas pseudoalcaligenes	0.9030
Blended	s__Pseudomonas alcaligenes	0.9122
Control	s__Agrobacterium sullae	0.9139
Control	s__Ruminococcus bromii	0.9250
Control	s__Bifidobacterium pseudolongum	0.9280
Control	s__Stenotrophomonas maltophilia	0.9481
Control	s__Raoultella terrigena	0.9697
Control	s__Profftia tarda	0.9761
Blended	s__Clostridium sartagoforme	0.9848
	**At genus level**	
Blended	g__Aggregatibacter	**0.0032**
Blended	g__Deinococcus	**0.0041**
Blended	g__Exiguobacterium	**0.0070**
Control	g__Geobacter	**0.0079**
Control	g__Thermomonas	**0.0253**
Control	g__Clostridium	**0.0263**
Control	g__Ramlibacter	**0.0294**
Control	g__Streptococcus	**0.0408**
Control	g__Herminiimonas	0.0533
Blended	g__Alcanivorax	0.0726
Blended	g__Pseudoramibacter_Eubacterium	0.0746
Control	g__WAL_1855D	0.0817
Blended	g__Lactonifactor	0.0854
Blended	g__Euzebya	0.0867
Control	g__Desulfovibrio	0.0981

Control	g__Gracilibacillus	0.0996
Control	g__Dokdonella	0.1014
Blended	g__Holdemania	0.1025
Blended	g__Octadecabacter	0.1097
Blended	g__Tolumonas	0.1146
Control	g__Xanthomonas	0.1149
Blended	g__Ruminobacter	0.1151
Control	g__Leptothrix	0.1158
Control	g__Succinimonas	0.1200
Control	g__Dorea	0.1254
Blended	g__Marinobacterium	0.1286
Control	g__Lacticigenium	0.1294
Control	g__Variovorax	0.1364
Control	g__Ochrobactrum	0.1387
Control	g__Thauera	0.1461
Blended	g__Bacteroides	0.1496
Control	g__Acetobacterium	0.1555
Blended	g__Selenomonas	0.1602
Blended	g__Acinetobacter	0.1631
Control	g__Leptospira	0.1671
Blended	g__Phascolarctobacterium	0.1684
Blended	g__Anaerotruncus	0.1756
Blended	g__Anoxybacillus	0.1760
Control	g__Parabacteroides	0.1812
Control	g__Coprococcus	0.1841
Blended	g__Cedecea	0.1901
Blended	g__Clostridium	0.1933
Blended	g__Lachnospira	0.1974
Blended	g__Salmonella	0.2002
Control	g__Gemella	0.2005

Blended	g__Faecalibacterium	0.2029
Blended	g__Methanobrevibacter	0.2063
Control	g__Anaerococcus	0.2077
Blended	g__Epulopiscium	0.2118
Blended	g__Eggerthella	0.2147
Control	g__Blautia	0.2215
Control	g__Alistipes	0.2234
Control	g__Sporosarcina	0.2247
Control	g__Thermicanus	0.2284
Blended	g__Paludibacter	0.2284
Blended	g__Paraprevotella	0.2326
Blended	g__Anaerovibrio	0.2392
Blended	g__Caloramator	0.2408
Control	g__Acinetobacter	0.2411
Blended	g__Odoribacter	0.2460
Control	g__Borrelia	0.2464
Blended	g__Thalassomonas	0.2553
Control	g__Pseudoxanthomonas	0.2554
Control	g__Leuconostoc	0.2586
Control	g__Sutterella	0.2589
Control	g__Alkalimonas	0.2609
Control	g__Acidaminococcus	0.2637
Blended	g__Adlercreutzia	0.2688
Control	g__Microbacterium	0.2692
Control	g__Jeotgalicoccus	0.2727
Control	g__Pelomonas	0.2745
Control	g__Achromobacter	0.2764
Blended	g__Gracilibacter	0.2789
Control	g__Parvimonas	0.2792
Control	g__Massilia	0.2859

Control	g__Limnohabitans	0.2923
Control	g__Burkholderia	0.2933
Control	g__Marinomonas	0.2977
Control	g__Succinivibrio	0.2984
Control	g__Lactobacillus	0.3033
Control	g__Abiotrophia	0.3090
Blended	g__human	0.3105
Blended	g__Roseburia	0.3114
Control	g__Arthrobacter	0.3116
Control	g__Turicibacter	0.3118
Control	g__Sarcina	0.3148
Control	g__Bulleidia	0.3183
Control	g__Rahnella	0.3221
Blended	g__Candidatus Azobacteroides	0.3306
Control	g__Mycoplana	0.3315
Blended	g__Tannerella	0.3323
Control	g__Megasphaera	0.3354
Control	g__Lachnobacterium	0.3380
Control	g__Limnobacter	0.3489
Control	g__Roseateles	0.3515
Control	g__Peptostreptococcus	0.3526
Control	g__Candidatus Phytoplasma	0.3542
Control	g__Fusibacter	0.3557
Control	g__Gluconacetobacter	0.3585
Control	g__Weissella	0.3595
Blended	g__Sodalis	0.3628
Control	g__Lactococcus	0.3676
Control	g__Stenotrophomonas	0.3728
Control	g__Arcobacter	0.3770
Blended	g__Pseudoalteromonas	0.3782

Blended	g__Enhydrobacter	0.3823
Blended	g__Staphylococcus	0.3836
Control	g__Pediococcus	0.3839
Control	g__Brevundimonas	0.3852
Control	g__Rummeliibacillus	0.3854
Control	g__Oceanobacillus	0.3870
Control	g__Psychrobacter	0.3925
Control	g__Flavobacterium	0.3933
Control	g__Collinsella	0.3949
Control	g__Robinsoniella	0.3956
Control	g__Sphingobacterium	0.3974
Control	g__Actinomyces	0.4038
Control	g__Methylibium	0.4045
Control	g__Rubrobacter	0.4050
Control	g__Acetobacter	0.4051
Control	g__Buchnera	0.4068
Control	g__Cellvibrio	0.4076
Control	g__Slackia	0.4135
Control	g__Comamonas	0.4138
Control	g__Mobiluncus	0.4139
Blended	g__Devosia	0.4171
Blended	g__Klebsiella	0.4178
Control	g__Finegoldia	0.4189
Control	g__Mycobacterium	0.4208
Control	g__Paucibacter	0.4218
Control	g__Desulfitobacter	0.4228
Control	g__Delftia	0.4249
Control	g__Peptoniphilus	0.4294
Control	g__Natronobacillus	0.4297
Blended	g__Pectinatus	0.4311

Control	g__Mogibacterium	0.4321
Blended	g__Desulfococcus	0.4399
Control	g__[Prevotella]	0.4414
Control	g__Sphingomonas	0.4421
Control	g__Rhodobacter	0.4444
Control	g__Pyramidobacter	0.4444
Control	g__Rhodopseudomonas	0.4444
Control	g__Fusobacterium	0.4444
Control	g__Oribacterium	0.4448
Control	g__[Ruminococcus]	0.4565
Blended	g__Kaistobacter	0.4586
Control	g__Porphyromonas	0.4599
Control	g__Micrococcus	0.4612
Control	g__Alcaligenes	0.4630
Control	g__Gallicola	0.4637
Control	g__Perlucidibaca	0.4641
Blended	g__Candidatus Hamiltonella	0.4658
Blended	g__Rhodobacter	0.4677
Blended	g__Vibrio	0.4698
Control	g__Trabulsiella	0.4708
Control	g__Nesterenkonia	0.4744
Control	g__Paenibacillus	0.4748
Control	g__Rhodoferax	0.4815
Control	g__Reinekea	0.4816
Control	g__Candidatus Accumulibacter	0.4872
Control	g__Serratia	0.4922
Blended	g__Schneideria	0.4926
Blended	g__Oscillospira	0.4933
Blended	g__Haloferula	0.4945
Control	g__Bifidobacterium	0.4953

Control	g__Clostridium	0.4957
Control	g__Corynebacterium	0.4983
Blended	g__Anaerofustis	0.4999
Control	g__Alicyclobacillus	0.4999
Control	g__Rhodoplanes	0.5023
Blended	g__Coprobacillus	0.5041
Blended	g__Photorhabdus	0.5078
Control	g__Lysinibacillus	0.5170
Control	g__Janthinobacterium	0.5206
Blended	g__[Clostridium]	0.5210
Blended	g__Enterobacter	0.5235
Control	g__Geodermatophilus	0.5255
Control	g__Aerococcus	0.5306
Control	g__Dechloromonas	0.5314
Control	g__Agrobacterium	0.5454
Control	g__Plesiomonas	0.5457
Control	g__Anaerobacillus	0.5478
Blended	g__Halorhodospira	0.5503
Control	g__Ralstonia	0.5530
Control	g__Polaromonas	0.5549
Blended	g__Shewanella	0.5580
Control	g__Yersinia	0.5595
Blended	g__Methylophaga	0.5606
Blended	g__Marinimicrobium	0.5632
Control	g__Ruminococcus	0.5655
Control	g__Enterococcus	0.5657
Control	g__Granulicatella	0.5720
Control	g__Acidithiobacillus	0.5720
Control	g__Clostridium	0.5730
Blended	g__Erwinia	0.5825

Blended	g__Candidatus Solibacter	0.5890
Control	g__Azorhizophilus	0.5892
Control	g__Halomonas	0.5899
Blended	g__Candidatus Phlomobacter	0.5945
Control	g__Trichococcus	0.6090
Control	g__Dysgonomonas	0.6093
Blended	g__Christensenella	0.6196
Blended	g__Moryella	0.6197
Control	g__Pseudidiomarina	0.6241
Control	g__Citrobacter	0.6249
Blended	g__Thalassiosira	0.6272
Blended	g__Pantoea	0.6303
Blended	g__Bilophila	0.6324
Control	g__Edwardsiella	0.6328
Blended	g__Dickeya	0.6362
Control	g__Vagococcus	0.6435
Control	g__Moraxella	0.6435
Control	g__Legionella	0.6472
Control	g__Carnobacterium	0.6491
Control	g__Filifactor	0.6595
Control	g__Actinobacillus	0.6605
Control	g__Treponema	0.6622
Control	g__Listeria	0.6675
Blended	g__Catenibacterium	0.6744
Control	g__Anaerostipes	0.6809
Control	g__Swaminathania	0.6855
Control	g__Bacillus	0.6868
Control	g__Proteus	0.6880
Control	g__Streptomyces	0.6917
Blended	g__Brenneria	0.6935

Control	g__Dictyostelium	0.6948
Control	g__Bombiscardovia	0.6949
Control	g__Solibacillus	0.7011
Control	g__Salinicoccus	0.7035
Control	g__Melissococcus	0.7037
Blended	g__Neisseria	0.7060
Control	g__Dehalobacterium	0.7062
Blended	g__Pseudomonas	0.7126
Blended	g__Geobacillus	0.7169
Control	g__Shigella	0.7249
Blended	g__Schwartzia	0.7289
Blended	g__Veillonella	0.7382
Control	g__Raoultella	0.7399
Blended	g__Bradyrhizobium	0.7453
Control	g__Desulfotomaculum	0.7457
Blended	g__Candidatus Blochmannia	0.7487
Control	g__Histophilus	0.7516
Blended	g__Kordia	0.7591
Blended	g__Oceanimonas	0.7614
Control	g__Methylobacterium	0.7620
Blended	g__Campylobacter	0.7628
Blended	g__Photobacterium	0.7675
Blended	g__Desulfovermiculus	0.7684
Control	g__Propionigenium	0.7738
Control	g__Nitrincola	0.7760
Blended	g__Candidatus Cardinium	0.7847
Blended	g__Anaerobaculum	0.7857
Control	g__Dialister	0.7872
Control	g__Haemophilus	0.7917
Blended	g__Leucothrix	0.7925

Blended	g__Moritella	0.7925
Blended	g__Fimbriimonas	0.8011
Control	g__Symbiobacterium	0.8028
Blended	g__Escherichia	0.8037
Blended	g__Akkermansia	0.8083
Control	g__Oleispira	0.8090
Control	g__Marinococcus	0.8198
Control	g__Zoogloea	0.8208
Blended	g__Acidaminobacter	0.8222
Blended	g__Megamonas	0.8232
Control	g__Morganella	0.8375
Control	g__[Eubacterium]	0.8388
Control	g__Bdellovibrio	0.8394
Blended	g__Butyrivibrio	0.8406
Blended	g__Kosmotoga	0.8432
Blended	g__Planococcus	0.8516
Control	g__Sporanaerobacter	0.8520
Control	g__Pseudobutyrivibrio	0.8520
Blended	g__Brevibacillus	0.8585
Control	g__Cronobacter	0.8605
Blended	g__Aeromonas	0.8613
Control	g__Asticcacaulis	0.8666
Control	g__Aquamonas	0.8697
Control	g__Polaribacter	0.8705
Blended	g__Providencia	0.8801
Control	g__Chryseobacterium	0.8834
Blended	g__Lysobacter	0.8869
Control	g__HTCC	0.8904
Blended	g__Gluconacetobacter	0.8963
Control	g__Halochromatium	0.9009

Control	g__Thermoanaerobacterium	0.9020
Blended	g__Clostridium	0.9051
Control	g__Nitrospira	0.9092
Blended	g__Marinobacter	0.9183
Blended	g__Allobaculum	0.9220
Blended	g__Desemzia	0.9235
Control	g__Alteromonas	0.9251
Control	g__Polynucleobacter	0.9254
Blended	g__Rheinheimera	0.9385
Blended	g__Proteiniclasticum	0.9392
Blended	g__Prevotella	0.9412
Control	g__Profftia	0.9485
Blended	g__Thiovirga	0.9500
Blended	g__Tatumella	0.9508
Control	g__Moellerella	0.9528
Blended	g__Butyricimonas	0.9535
Blended	g__Salinispora	0.9680
Control	g__Leclercia	0.9712
Control	g__Chelonobacter	0.9717
Blended	g__Alloiococcus	0.9723
Control	g__Glaciecola	0.9731
Blended	g__Succiniclasticum	0.9751
Control	g__Gordonia	0.9779
Control	g__Thiothrix	0.9782
Control	g__Desulfosporosinus	0.9797
Control	g__Facklamia	0.9838
Control	g__Shuttleworthia	0.9840
Blended	g__Aequorivita	0.9872
Control	g__Novosphingobium	0.9888
Control	g__Candidatus Regiella	0.9898

Blended	g__Candidatus Portiera	0.9909
Blended	g__Rothia	0.9911
Blended	g__Ferrimonas	0.9969
	**At Phylum level**	
Control	p__Spirochaetes	**0.0321**
Control	p__Planctomycetes	0.1860
Blended	p__Euryarchaeota	0.2064
Blended	p__Acidobacteria	0.2825
Blended	p__Bacteroidetes	0.3546
Blended	p__Tenericutes	0.4218
Control	p__Gemmatimonadetes	0.4305
Control	p__Fusobacteria	0.4371
Blended	p__Armatimonadetes	0.4919
Control	p__Actinobacteria	0.4985
Control	p__Synergistetes	0.5120
Control	p__Firmicutes	0.5497
Control	p__Elusimicrobia	0.5628
Control	p__Fibrobacteres	0.6199
Control	p__Verrucomicrobia	0.7568
Control	p__Cyanobacteria	0.7588
Control	p__Chloroflexi	0.7625
Blended	p__Crenarchaeota	0.9032
Blended	p__Thermotogae	0.9060
Blended	p__Nitrospirae	0.9170
Blended	p__Proteobacteria	0.9986

Supplemental Table S3. Richness, Shannon Diversity Index (SDI) and Evenness across BTFs, Elemental and Polymeric formulas. No statistical significance noted among any of the indices.

**Mean value**	**Shannon's diversity index**	**Richness (no. of OTUs)**	**Pielou's Evenness Index**
**At species level**			
BTF (11)	2.44	115	0.52
Polymeric (11)	2.38	118	0.51
Elemental (7)	2.21	112	0.47
BTF vs polymeric (P-value)	0.82	0.78	0.65
BTF vs elemental	0.47	0.88	0.42
Polymeric vs elemental	0.40	0.72	0.29

**At genus level**			
BTF (11)	2.11	184	0.40
Polymeric (11)	2.16	188	0.42
Elemental (7)	2.46	180	0.48
BTF vs polymeric (P-value)	0.13	0.83	0.10
BTF vs elemental P-value	0.83	0.84	0.77
Polymeric vs elemental P-value	0.23	0.74	0.24
